# Common bean SNP alleles and candidate genes affecting photosynthesis under contrasting water regimes

**DOI:** 10.1038/s41438-020-00434-6

**Published:** 2021-01-01

**Authors:** Susana Trindade Leitão, Maria Catarina Bicho, Priscila Pereira, Maria João Paulo, Marcos Malosetti, Susana de Sousa Araújo, Fred van Eeuwijk, Maria Carlota Vaz Patto

**Affiliations:** 1 Instituto de Tecnologia Química e Biológica António Xavier, Universidade Nova de Lisboa, Oeiras, Portugal; 2 Wageningen University & Research, Wageningen, The Netherlands; 3 Present Address: Instituto Superior de Agronomia, Universidade de Lisboa, Lisboa, Portugal; 4 Present Address: Nunhems Vegetable Seeds, Nunhem, The Netherlands; 5 Present Address: Association BLC3—Technology and Innovation Campus, Centre Bio R&D Unit, Oliveira do Hospital, Lisboa, Portugal

**Keywords:** Natural variation in plants, Abiotic, Plant genetics

## Abstract

Water deficit is a major worldwide constraint to common bean (*Phaseolus vulgaris* L.) production, being photosynthesis one of the most affected physiological processes. To gain insights into the genetic basis of the photosynthetic response of common bean under water-limited conditions, a collection of 158 Portuguese accessions was grown under both well-watered and water-deficit regimes. Leaf gas-exchange parameters were measured and photosynthetic pigments quantified. The same collection was genotyped using SNP arrays, and SNP-trait associations tested considering a linear mixed model accounting for the genetic relatedness among accessions. A total of 133 SNP-trait associations were identified for net CO_2_ assimilation rate, transpiration rate, stomatal conductance, and chlorophylls *a* and *b*, carotenes, and xanthophyll contents. Ninety of these associations were detected under water-deficit and 43 under well-watered conditions, with only two associations common to both treatments. Identified candidate genes revealed that stomatal regulation, protein translocation across membranes, redox mechanisms, hormone, and osmotic stress signaling were the most relevant processes involved in common bean response to water-limited conditions. These candidates are now preferential targets for common bean water-deficit-tolerance breeding. Additionally, new sources of water-deficit tolerance of Andean, Mesoamerican, and admixed origin were detected as accessions valuable for breeding, and not yet explored.

## Introduction

Common bean (*Phaseolus vulgaris* L.) is one of the most important food-grain legumes worldwide, with recognized benefits for health and nutrition^1^. Water availability is the major abiotic factor affecting crop productivity. Drought periods may result in up to 70% of yield reduction^2^. It is estimated that 60% of common bean production occurs in regions prone to water deficit^3^.

Under water deficit, many physiological processes, including photosynthesis, are negatively affected. Most plants respond to a mild-to-moderate water deficit by closing stomata and reducing carbon assimilation, limiting photosynthesis^4,5^. Stomata closure also leads to excess energy that, if not dissipated as heat, may be harmful to photosystem II through the production of reactive oxygen species (ROS)^6^. The cellular antioxidative and photoprotective defenses conferred by pigments, such as carotenoids, may scavenge these ROS^7^. Leaf photosynthetic pigment (chlorophyll *a*, C*a*, and chlorophyll *b*, C*b*) content and the chlorophyll *a*/*b* ratio may also be affected by water deficit, depending on the species and genotype^8–10^.

It is known that the two common bean gene pools—Andean and Mesoamerican—differ in their molecular, agronomic, morphological, and physiological characteristics, including the mechanisms by which common bean tolerates water deficit^11^. As an example, the identification of drought-tolerant sources has been achieved within the Mesoamerican gene-pool race Durango^3,12^, whereas only a few sources of tolerance were identified in the Andean gene pool^13^.

Understanding the mechanisms underlying water-deficit tolerance is of primary importance for devising precision-breeding approaches. Quantitative trait loci (QTL) mapping studies using common bean recombinant inbred lines (RIL) have been used to identify loci associated with water-deficit tolerance and yield-component traits^14–21^. With the release of the *P. vulgaris* L. genome^22^, genome-wide association studies (GWAS) have become the approach of choice in genetic research. Under the scope of these GWAS, accessions from both gene pools were screened for production traits, such as biomass, 100-seed weight, days to flower and maturity, and SPAD measurements under water deficit^23,24^. Nevertheless, the genetic basis of the photosynthetic response to water deficit is still not comprehensively understood in common bean.

The Portuguese common bean germplasm results from more than 500 years of natural and farmers’ selection. Genetically closer to the Andean gene pool, this germplasm also presents accessions of admixed (Andean × Mesoamerican) origin^25^. Due to this, this germplasm may contain unique genetic combinations that may circumvent the challenge of finding resistance sources useful for breeding within both gene pools. Still, the existence of water-deficit-tolerance sources within this germplasm is unknown since its performance was never characterized under water-deficit conditions.

To identify mild water-deficit-tolerant sources, and genomic regions or candidate genes associated with the natural variation of common bean photosynthetic response under contrasting water regimes, we phenotyped 158 diverse Portuguese common bean accessions under well-watered and water-deficit conditions using photosynthesis-related parameters. A GWAS combining these phenotypes with previously collected genotypic data was performed. This study will enable the development of molecular tools to increase the efficiency of common bean breeding for tolerance to water deficit.

## Results

### Phenotypic trait variation under contrasting water treatments

Several physiological and morphological traits were evaluated in the Portuguese common bean collection under well-watered (WW) and water-deficit (WD) conditions, namely, stomatal CO_2_ conductance (gs), net CO_2_ assimilation rate (A), transpiration rate (E), substomatal CO_2_ concentration (C*i*), instantaneous and intrinsic water-use efficiencies (WUE = A/E and WUE_i_ = A/gs, respectively), chlorophylls a (C*a*) and b (C*b*) contents, carotene and xanthophyll (C*cx*) contents, leaf fresh weight (FW), turgid weight (TW) and dry weight (DW), leaf relative water content (RWC (%) = [(FW − DW)/(TW − DW)] × 100), specific leaf area (SLA), and leaf thickness (LT).

High variability of phenotypic responses under WW and WD was observed within the Portuguese common bean collection (Supplementary Fig. S1). For the majority of the traits, SER16 (WD resistant) and Tio Canela-75 (WD sensitive) were intermediate to the Portuguese accession variation, with few exceptions. Both Mesoamerican references showed lower FW/DW and SLA, and higher LT than the Portuguese accessions. Tio Canela-75 had A values close to SER16 under WW. Nevertheless, Tio Canela-75 showed a stronger decrease in A upon WD.

Variance component estimation using a linear mixed model was performed to examine the effect of common bean accession, gene pool, and water treatment in the observed trait variation (Supplementary Table S2). Differences were detected between water treatments and the gene pool of origin (Andean, Mesoamerican, or admixed) (*P* value ≤ 0.05) for the majority of the traits, with the exception of FW/DW, C*a*/C*b*, and (C*a* + C*b*)/C*cx*, and also C*i* and SLA just in the case of the gene pool. On the other hand, the effect of the accession was significant for the majority of the traits with the exception of RWC, E, gs, C*a* + C*b*, and (C*a* + C*b*)/C*cx*. Treatment × accession interaction was significant for FW/DW, and for both WUEs, and treatment × gene pool of origin for FW/DW, E, gs, and WUEs (Supplementary Table S2). Differences between gene pools were mainly observed under WW. Under these conditions, the Portuguese accessions of Mesoamerican origin had higher (*P* value < 0.05) mean values of E and gs than the accessions more related to the Andean gene pool (Supplementary Table S3).

Gas-exchange parameters (A, E, gs, and C*i*) and RWC decreased with the soil water content reduction to 40% of FC. In contrast, C*a*, C*b*, C*cx*, and the WUEs increased under WD (Supplementary Table S2).

Trait broad-sense heritability estimates (*H*^2^) were in general below 0.5 and higher under WW than under WD, with the genetic variance components for the accessions following the same trend (Supplementary Table S4).

### Correlation between traits

**Fig. 1:**
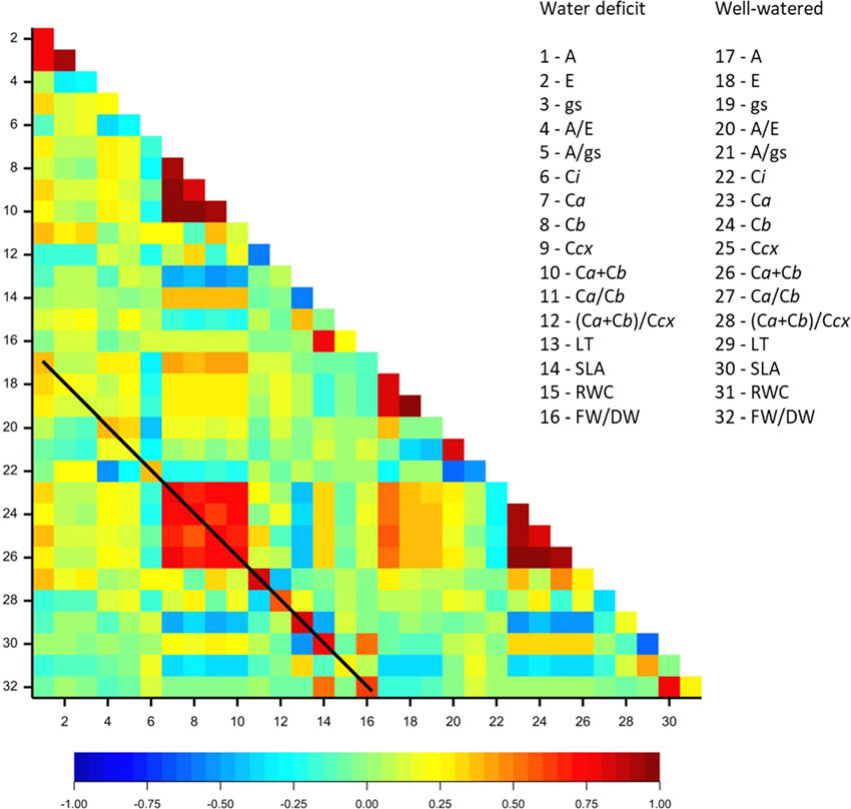
Correlation matrix heatmap with Pearson coefficients based on BLUEs for 160 common bean accessions, colored by a spectrum from blue (correlation = −1.0) to red (correlation = 1.0). The 16 traits under well-watered and water-deficit conditions are shown. A black line crosses along the diagonal containing the pairwise correlations of the same traits under both water treatments (autocorrelations)

Two groups of highly correlated traits (Pearson’s correlation >0.75) under both water treatments were detected: the A, E, and gs gas-exchange measurements and the C*a*, C*b*, and C*cx* leaf-pigment content (Fig. 1).

Traits related to leaf morphology and water content (SLA and FW/DW) were also highly correlated (*r* = 0.80 under both treatments). Leaf pigments and leaf-morphology traits showed the highest autocorrelation between water treatments. The correlation matrix between traits is available in Supplementary File S5.

### Accession phenotypic relatedness

**Fig. 2:**
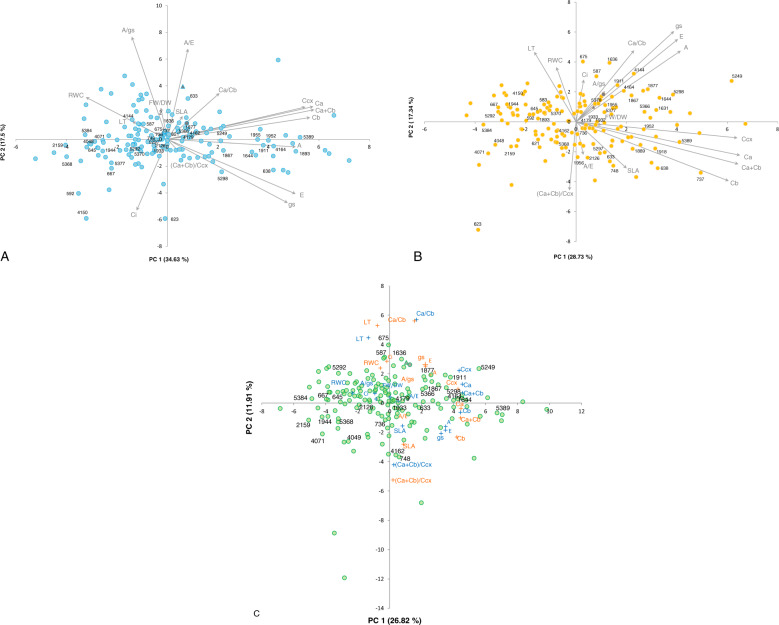
Principal component analysis based on the BLUE values for 16 phenotypic traits, using 160 common bean accessions (158 Portuguese, SER16, and Tio Canela-75) under well-watered and water-deficit conditions. Biplots are shown separately for well-watered (**A**), and water-deficit (**B**) conditions, with the accessions represented in blue and orange, respectively, and trait loading vectors in gray. In **C**, the loadings of the 16 traits under both water treatments are represented together (well watered in blue, and water deficit in orange), and the accessions are represented in green. The Mesoamerican lines SER16 and Tio Canela-75 are represented in gray by a circle or triangle, respectively. Accession numbers depicted are the ones mentioned throughout the text

A PCA was conducted with the 16 traits evaluated under WW and WD conditions and biplots generated (Fig. 2). Figure 2A (WW) and 2B (WD) displays the 160 common bean accessions (158 Portuguese, SER16, and Tio Canela-75) scattered in the space defined by the first two components from the *Eigenanalysis*. The first two principal components explained 52% and 46% of the variance observed under WW and WD, respectively. Loading vectors of C*a*, C*b*, C*cx*, and C*a* + C*b* grouped together indicating a similar information contribution, irrespective of the water treatment. The same occurred for the gas-exchange traits A, E, and gs. Under WW (Fig. 2A), the RWC vector pointed out in the opposite direction of E and gs vectors. Under WD (Fig. 2B), RWC was negatively correlated with WUE and SLA. Figure 2C displays in the same plot the 160 accessions and the 16 traits measured under the two water treatments. The first two principal components explained almost 39% of the variance observed. Accessions with high A, regardless of the water treatment, were identified (e.g., 587, 1877, 1911, 4164, 5298, 5366, and 5389 of Andean origin, 1636, 1644, 1867, 5249, and SER16 of Mesoamerican origin, and 675 of admixed origin). From those, the ones with higher WUEs under WD were 587, 5366, and 5389.

**Fig. 3:**
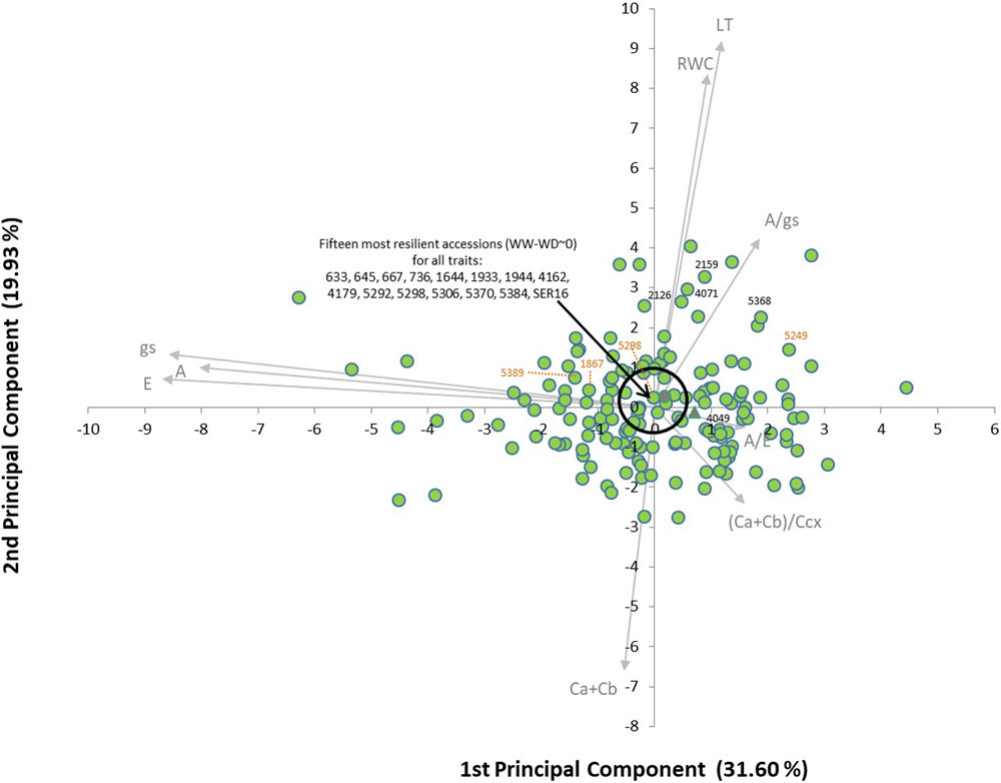
Principal component analysis based on the differences observed between the BLUE values under well-watered and water-deficit conditions of 9 phenotypic traits, for 158 Portuguese common bean accessions, and the Mesoamerican lines SER16 (represented by a gray circle) and Tio Canela-75 (represented by a gray triangle). The first two components explained 51.53% of the observed variation. Accessions inside the black circle are the ones for which all the traits varied the least between water treatments, and were considered the most resilient. Highlighted accessions had high A values (in orange) and high WUE (A/E, in black) under both conditions, but also a relatively high leaf RWC variation between water treatments

To identify water-deficit-resilient accessions, an extra PCA was performed (Fig. 3) using the differences between the BLUEs obtained for each trait under WW and WD. This allowed the detection of accessions with little variation under both water treatments (the ones closest to the graph-axis origin), considered more resilient. Since some trait vectors were redundant, only 9 out of the 16 traits are shown (C*a* + C*b*, (C*a* + C*b*)/C*cx*, A, E, gs, A/E, A/gs, LT, and RWC).

The 15 most resilient accessions were highlighted by a central black circle (Fig. 3) and are summarized in Table 1, together with other interesting accessions that, for the traits evaluated, revealed a good performance or increased photosynthetic pigment content under water-deficit conditions.

**Table 1:** Summary of the Portuguese common bean accessions with the most interesting responses to water-deficit conditions, with the indication of the gene pool of origin

	Origin of accessions		
	Andean	Mesoamerican	Admixed
Most resilient^a^ accessions	633, 645, 667, 736, 1933, 1944, 4179, 5298, 5384	1644, 5292, 5370, and SER16	4162
Accessions with both small A and RWC variations between treatments	587	1636	675
Accessions with small A and large RWC variations between treatments	5298, 5389	1867, 5249	
Accessions with small WUE and large RWC variations between treatments	4048	1932, 1955	
Accessions with increased WUE and decreased RWC under WD	621, 633, 700		
Accessions with the highest increase in C*a* + C*b* under WD	737	748, 5249	5368

^a^Most resilient accessions were the ones for which all the traits measured varied the least when comparing well-watered and water-deficit conditions

### Genomic regions associated with photosynthesis-related traits

The trait-adjusted means were tested for association with 9,825 SNP markers, obtained from Illumina Infinium BARCBean6K_3 BeadChip^TM^ assay and DArTseq^TM^ analysis, scored in 144 common bean accessions that passed the genotypic and phenotypic quality filters applied.

**Fig. 4:**
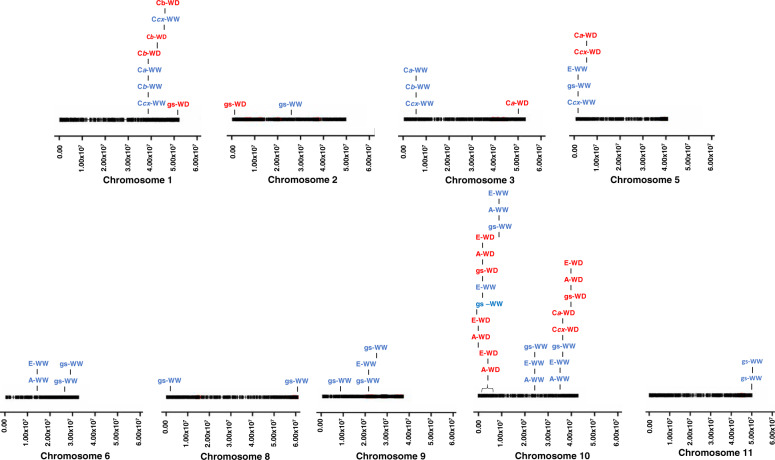
Schematic representation of the common bean chromosomal regions significantly associated with the traits A, E, gs, Ca, Cb, and Ccx, under well-watered (in blue) and water-deficit (in red) conditions, using 144 Portuguese accessions (the ones fulfilling genotypic quality filtering). Traits in the same column were associated with the same SNP markers. Horizontal bars represent common bean chromosomes and SNP position indicated in base pairs, based on the *Phaseolus vulgaris* genome v2.1. Only chromosomes with significant marker-trait associations are depicted

A total of 133 significant marker-trait associations were identified, 43 under WW and 90 under WD (threshold set as –log_10_ (*P* value) = 3), for the six traits studied (Supplementary File S6). Of those, 112 were related to gas-exchange parameters (A, E, and gs), while the remaining 21 were related to leaf-pigment contents (C*a*, C*b*, and C*cx*). The marker-trait associations were scattered throughout the chromosomes, except for chromosomes 4 and 7, where no significant associations were identified (Fig. 4). Chromosome 10 was the one with more SNP-trait associations detected: apart from C*b*, all trait variations were associated with this chromosome. Only two SNPs were associated with the same trait under both water treatments. This was the case of SNP00315 (at 37.69 Mbp in chromosome 1) associated with C*b*, and of DART09339 (at 3.84 Mbp in chromosome 10), associated with E (Supplementary File S6 and Fig. 4).

#### Regions with the strongest marker-trait associations

**Fig. 5:**
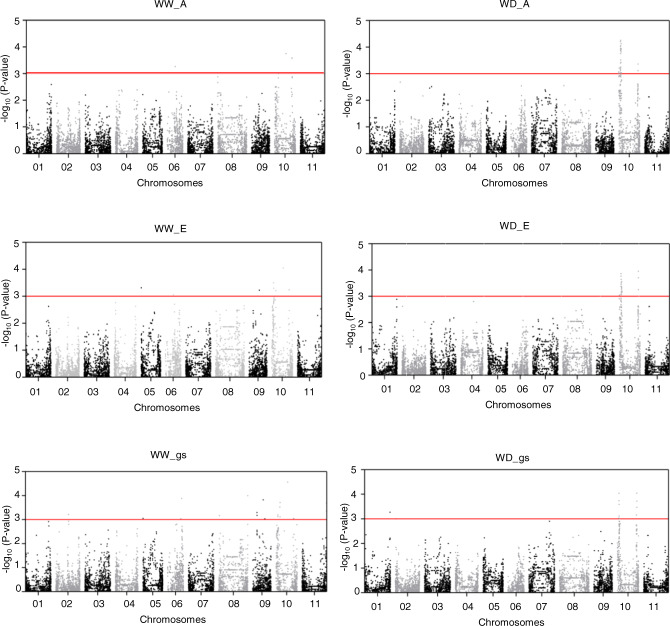
Manhattan plot depicting the genome-wide association results for net CO_2_ assimilation rate (A), transpiration rate (E), and stomatal conductance of CO_2_ (gs), in 144 Portuguese common bean accessions, under well-watered (WW, on the left) and water-deficit (WD, on the right) conditions. The *y* axis represents the −log_10_ (*P* value) of 9,825 SNPs, and the *x* axis shows their chromosomal positions across the common bean genome. The horizontal red line indicates the significance threshold (*P* value = 1 × 10^−3^)

Under WD, the strongest marker-trait associations (4.03 < −log_10_ (*P* value) < 4.25) were detected in chromosome 10, between 3.31 and 4.76 Mbp, for A, E, and gs (Fig. 5 and Supplementary File S6). Under WW, SNP01123 (in chromosome 3, at 5.30 Mbp) was strongly associated with C*a* and C*b* (−log_10_ (*P* value) = 4.82, the strongest association identified), while SNP04526 (in chromosome 10, at 27.9 Mbp) was strongly associated with E and gs (−log_10_ (*P* value) = 4.05 and 4.56, respectively) (Fig. 5 and Supplementary Figs. S2–S4).

#### Regions with multiple-trait associations

Interestingly, 75 SNPs were responsible for 133 marker-trait associations detected. This indicates that, frequently, the same SNP was associated with different traits.

Under WW conditions, SNP04526, SNP04627, and SNP04633 were associated with both A and E, in chromosome 10 (27.88, 36.89, and 37.28 Mbp, respectively). Under WD conditions, another region of the same chromosome (3.16–5.96 Mbp) contained 29 SNPs associated with A and 22 SNPs with E (Fig. 4). Another example is SNP00315, in chromosome 1 (37.69 Mbp), associated with C*a*, C*b*, and C*cx*, under WW and with C*b* under WD.

The complete list of marker-trait associations with marker names, genome positions, effect and frequency of the variant allele, and −log_10_ (*P* value) is available in Supplementary File S6.

The Manhattan plots depicting the GWAS results for A, E, and gs, under WW and WD, are shown (Fig. 5). The Manhattan plots for C*a*, C*b*, and C*cx* are available in Supplementary Figs. S2–S4.

Chromosome 10 gathered most of the marker-trait associations. At this chromosome, and under WW, four SNPs were associated with A, six with E, and eight with gs. Under WD, and at the same chromosome, 36 SNPs were associated with A, 28 with E, and 11 with gs.

### Effect of variant allele and proportion of variance explained by each SNP-trait association

The effect of the variant allele was positive in almost all the SNPs associated with the traits under WD (Supplementary File S6). Under this water treatment, the variant allele had a negative effect only in three SNPs: DART01093 associated with gs (variant allele effect = −0.0363), DART03370 associated with C*a* (variant allele effect = −0.2721), and SNP00315 associated with C*b* (variant allele effect = −0.0888). Under WW, the effect of the variant allele was positive for 29 out of 35 (82.9%) SNPs associated with A, E, and gs, and negative for 7 out of 8 (87.5%) SNPs associated with C*a*, C*b*, and C*cx*.

For all the traits, each SNP-trait association only explained a small portion of the observed phenotypic variance (3.92–14.2%). The trait with the largest proportion of variance explained by an associated SNP was C*b* under WD (14.2%), followed by C*a* under WD (12.4%), and gs under WW (12.0%) (Supplementary Table S7).

### SNP allelic variant frequency among gene pools of the origin of accessions

The frequency of the variant allele in 75 associated SNPs was different among the gene pool of origin (Supplementary Fig. S5). On average, the accessions of Mesoamerican origin had a higher frequency of the variant allele than the accessions of Andean and admixed origin. The average frequency of the variant allele in the accessions of admixture origin was in most cases intermediate between the accessions of Andean and Mesoamerican origin.

### Candidate gene identification

The genomic locations of the SNPs associated with the traits were inspected using the *JBrowse* tool in *P. vulgaris* v2.1 genome and 95 positional or LD candidate genes were identified. In brief, from the 90 SNPs found associated with the traits under WD, 66.7% mapped within genes or were in LD with SNPs located within candidate genes. On the other side, from 43 SNPs associated with the traits under WW, 60.5% mapped within genes or were in LD with SNPs located within candidate genes. A mapping resolution to the gene level was achieved in 55% of the cases, with a single gene identified within the LD block around the associated SNP. A complete list of the candidate genes, their functional annotation, and putative role in controlling the traits under scrutiny can be found in Supplementary File S6.

### Gene-trait network analysis and functional categorization of candidate genes

Molecular-interaction networks were established between 95 candidate genes associated with the traits, under WW and WD (Supplementary Fig. 6).

Most of the interconnections were established between the gas-exchange parameters (A, E, and gs) under WD conditions. A bridge between the two water treatments was detected for A-WD and E-WD, and E-WW through the gene Phvul.010G026100. This gene encodes for a disease-resistance protein (TIR–NBS–LRR class) family and was functionally categorized as “External stimuli response”. Also, Phvul.010G023500, encoding for a disease-resistance protein (TIR–NBS–LRR class) family, was found to bridge E-WD, gs-WD, and A-WD. Both genes contained the strongest associated SNPs. Regarding pigment contents, two different clusters were obtained reflecting the treatments applied. Importantly, the networks evidenced a concerted action of multiple genes controlling the traits studied, especially under WD conditions.

**Fig. 6:**
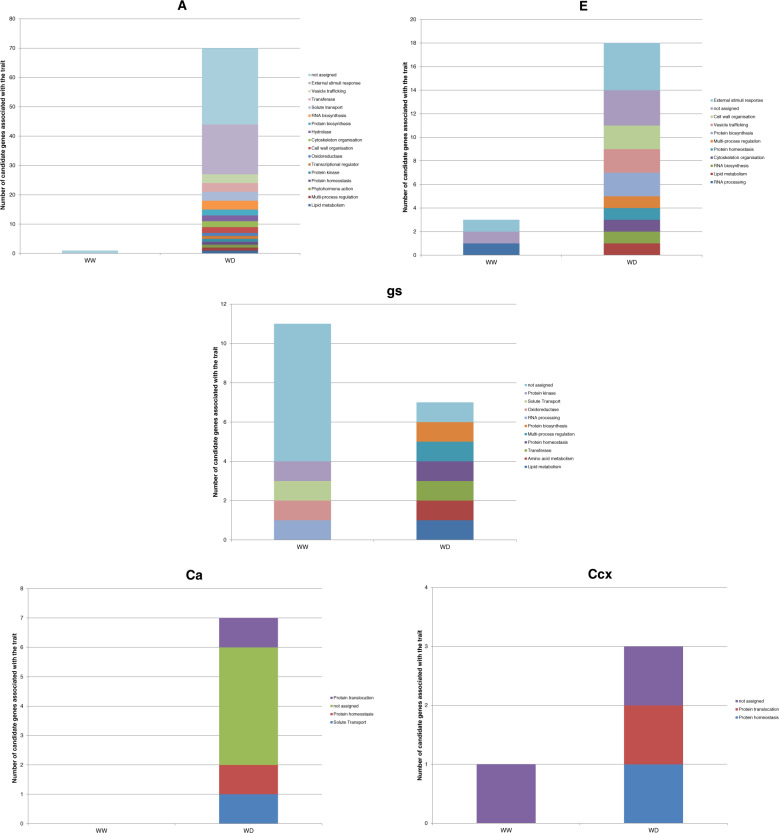
MapMan functional categories of the candidate genes associated with each trait under well-watered (WW) and water-deficit (WD) conditions. The five bar charts represent the number of candidate genes of a given functional category associated with A, E, gs, Ca, and Ccx

The MapMan functional categorization of the candidate genes supported the existence of interactions between genes acting in different metabolic pathways. Under WW, the functional categories of the assigned genes were “Protein kinase”, “External stimuli response”, “Solute transport”, “Oxidoreductase”, and “RNA processing”, with 7% of frequency each. Under WD, the assigned genes were allocated to 18 functional categories, with “External stimuli response” (20%), “Transferase” (5%), “Solute transport” (5%), “Vesicle trafficking” (4%), and “RNA biosynthesis” (4%), the most frequent ones (Fig. 6, and Supplementary Table S8 for bin names and codes of all genes).

In the frame of this work, it was not possible to describe all putative candidate genes located within the associated genomic regions in detail. Therefore, in Supplementary Table S8, we highlighted the candidate genes located in regions where the strongest associations were detected, associated with multiple traits, and the ones that we considered to have a more relevant biological function.

## Discussion

Drought is a major concern in agriculture affecting a wide range of crops, including common bean. The capacity of plants to adapt to water deficit and prevent its negative impact on growth and reproduction is related to the plasticity and resilience of the photosynthetic process^26^. The genetic basis of photosynthesis-related traits controlling plant response under well-watered and water-deficit conditions is still not totally understood in common bean. In this context, this study characterized for the first time the natural variation in leaf morphology, pigment contents, and photosynthesis-related traits found in a collection of 158 Portuguese common bean accessions. This Portuguese collection is known for its genetic admixture between the original Mesoamerican and Andean gene pools^25^. Additionally, and using a GWAS approach, a total of 133 common bean genomic regions controlling the natural variation found for photosynthetic performance-related traits (gas exchange and pigment contents) under contrasting water conditions were identified.

Accessions with higher CO_2_ assimilation rate (A), water-use efficiencies (WUEs), and pigment contents under water deficit were highlighted within each gene pool, and SNP markers and candidate genes associated with this more resilient photosynthetic performance identified. Moreover, the complementary evaluation of photosynthesis-related traits under well-watered conditions allowed the identification of accessions with higher A and WUEs suited to be grown in regions where water deprivation is not a constraint. This knowledge provides an opportunity to develop novel molecular tools to sustain a more effective selection of water-responsive common bean germplasm adapted to different environments.

### A diversity of physiological responses to water deficit

The leaf RWC decreased on average less than 10% under WD, indicating that leaf water status did not change severely when the soil water content was reduced to 40% of FC. Nevertheless, this was enough to trigger relevant physiological responses. As expected, a general reduction in the photosynthesis-related parameters (A, E, and gs) was observed under WD. The decline of photosynthetic and transpiration rates and the closure of the stomata are among the most frequent responses of plants facing WD^27^. Considering the entire common bean collection, A, E, and gs decreased on average 46%, 56%, and 71% under WD. Stomatal conductance (gs) was the photosynthetic parameter most affected by the imposed water deprivation. This confirms that common bean plants subjected to WD activated the mechanisms that allow water retention, as the first line of defense against water scarcity. Indeed, this behavior has been reported in many studies describing C3 plant response under mild-to-moderate drought^28^, including common bean^13,29–31^.

The Portuguese collection presented a large variability of photosynthetic responses to the studied conditions. For instance, we identified accessions with high A values both in WW and WD. These accessions can be considered resilient to the tested WD conditions, maintaining a good photosynthetic performance (A values) even under stress. Among those accessions, 587, 675, 1636, 5249, and 5298 had a better A under WD conditions than, for example, SER16, an elite drought-tolerant CIAT accession. We also identified accessions, such as 623, 638, 1893, and 1918, with the highest A values under WW, but very susceptible under WD. These accessions are more adequate for irrigated farming systems. Interestingly, we also found accessions in which WD enhanced their photosynthetic performance such as 592, 4144, 4150, and 5377.

Despite the general negative effect of WD on gas-exchange parameters, most accessions displayed improved WUEs under WD when compared to WW. Indeed, in our study, a significant accession × treatment interaction for WUEs was observed. This feature was already described for other bean cultivars under WD^31^. In response to WD, low stomatal conductance avoids water losses and increases water-use efficiencies^32^. However, the closure of the stomata also prevents CO_2_ from entering the leaf and, consequently, photosynthetic carbon assimilation is decreased in favor of photorespiration. The selection of accessions with higher WUEs, with a reduced need for water irrigation, has been considered essential to breed for drought-tolerant bean cultivars, maximizing crop yield in a more sustainable manner^3,13,33,34^. Following this selection criteria, common bean cultivars, mainly of Mesoamerican origin, with superior drought tolerance, have been identified^35^. Among the Portuguese germplasm, several accessions with high WUE values under the applied WD conditions were identified. In particular, accessions 587, 5366, and 5389 of Andean origin stood out, for having both high A and WUE values under WD. Interestingly, accession 587 was among the most resilient, with high A and little changes in leaf RWC upon WD, while accession 5389 had high A values maintained in WD, despite the decrease of its RWC. This is important information since few common bean sources of water-deficit tolerance of Andean origin had been identified until now and they are lacking especially for the improvement of bush-type beans usually cultivated in dry environments^3^.

Chlorophylls *a* and *b*, and carotene and xanthophyll contents increased, on average, 20% among the Portuguese accessions under WD. Few exceptions occurred, in which a decrease in these contents was observed in response to WD. As an example, accessions 1867, 2126, and 5297 of Mesoamerican, Andean, and admixed origin, respectively, showed a decrease in C*a* + C*b* in response to decreased RWC. Reports of common bean field studies also described a reduction in chlorophyll content in response to WD, due to the damage in chloroplasts caused by the formation of ROS species such as O_2_^–^ and H_2_O_2_ (refs. ^30,36^) and this is probably occurring also on these Portuguese accessions.

Regarding carotenoids, accessions 748, 1889, and 2159 of Mesoamerican, Andean, and admixed origin, respectively, showed an increase in their total content in response to the studied WD. Carotenoid accumulation and interconversion is a well-described strategy to counteract the negative effects of oxidative damage caused by the accumulation of ROS derived from the excess of light- excitation energy^27^. Indeed, their accumulation can be part of the strategy of these common bean accessions to cope with WD.

### Candidate loci and genes associated with photosynthetic performance

Several of the SNP-trait associations detected in the present study were located within or near a priori candidate gene involved in the water-deficit response, which strengthened and validated the usefulness of the Portuguese association panel. The SNPs with greater potential for an effective marker-assisted selection will be the ones strongly associated with a trait and for which the SNP variant allele has a higher effect on the trait variation. Accordingly, strong SNP-trait associations were identified that could result in an improvement of 19% of net CO_2_ assimilation rate, 8% of stomatal conductance, 16% of chlorophyll *a* concentration, and 14% of chlorophyll *b* concentration, under water-deficit conditions.

For the great majority of the detected associations with A, E, gs, C*a*, C*b*, and C*cx*, the variant allele had a positive effect, increasing the trait value. On the other hand, the average frequency of the variant allele varied according to the accession origin. For most of the associated SNP, the accessions of Mesoamerican origin had higher frequencies of the variant allele. This might reflect background-selection events during domestication and breeding history, with selection and fixation of alleles involved in water-deficit responses due to adaptation to different environmental ecosystems in Mesoamerica (in general more prone to drought scenarios) versus the Andes^3^.

Several markers were simultaneously associated with different gas-exchange parameters reflecting the high correlation existing between those traits. Some of the candidate genes identified as simultaneously associated with A, E, and gs, in chromosome 10, under water-deficit conditions, provided clues on the mechanisms of common bean activation to overcome water deficit. For example, the identified candidate gene Phvul.010G125000 encodes for a polyphosphoinositide phosphatase that in *Arabidopsis thaliana* is associated with an increased water-deficit tolerance due to the reduction of water loss^37^. The water-retaining capacity is essential in water-deficit-avoidance and -tolerance mechanisms. Moreover, four other identified candidate genes (Phvul.010G025100, Phvul.010G031700, Phvul.010G032000, and Phvul.010G032700) have functional annotations that reflect common bean responses to water deficit. Phvul.010G025100 codes for a NB-ARC leucine-rich repeat (LRR)-containing domain disease-resistance protein. The LRR-containing domain is evolutionarily conserved in many proteins associated with innate immunity in plants and has been implicated in diverse signaling events, including the ones involved in the early steps of osmotic stress regulation^38,39^. Indeed, the candidate gene-trait network analysis performed in the present work also evidenced other disease-resistance proteins with LRR domains encoded by Phvul.010G026100 and Phvul.010G023500 associated with more than one trait/treatment studied. On the other hand, Phvul.010G031700 codes for a gamma-tubulin complex component 2, a cytoskeleton protein described as drought-responsive and implicated in cell growth^40^. Additionally, Phvul.010G032000 codes for a receptor-like serine/threonine-protein kinase 1, and this class of proteins has known roles in signaling, development regulation, and plant defense^41^. For instance, the SnRK2 family members are plant-specific serine/threonine kinases involved in plant response to abiotic stresses and abscisic acid (ABA)-dependent signaling^42^. Finally, Phvul.010G032700 codes for a zinc finger protein involved in the transcriptional regulation responsive to abiotic stresses through the induction of the phytohormone ABA^43^. ABA is known to accumulate in cells and to be very abundant under water-deficit conditions, inducing the expression of many stress-related genes^44^. Moreover, ABA controls stomatal aperture through the biochemical regulation of ion- and water-transport processes^45^.

In the particular case of stomatal CO_2_ conductance (gs), the identified candidate gene Phvul.001G259400, under water-deficit conditions, encodes for a 3-oxoacyl-[acyl-carrier-protein] reductase that catalyzes the first reduction step in fatty acid biosynthesis and is repressed by ABA in guard cells of *A. thaliana*^46^. Also, Phvul.010G118300, associated with gs under WD, encodes a calcineurin B-like (CBL) interacting protein kinase. This class of proteins plays an important role in stress-signaling transduction, regulating Mg^2+^ and K^+^ homeostasis, and enhancing stress tolerance, for example in *A. thaliana* and rice^47^. Additionally, the identified candidate gene Phvul.010G024800, for net CO_2_ assimilation rate (A) under water-deficit conditions, encodes for a sterol-regulatory element-binding protein described as having a role in abiotic stress signaling in the endoplasmic reticulum of *A. thaliana*^48^.

Regarding the SNP associations with leaf pigments under water-deficit conditions, two candidate genes were identified for both C*a* and C*cx*: Phvul.005G045500 and Phvul.005G045600. The first encodes an aspartyl protease and the second an YT521-B-like domain. The relation of aspartic protease to drought susceptibility was found in *P. vulgaris* leaves by analyzing drought-tolerant and -susceptible plants that differed regarding the aspartic protease precursor gene expression^49,50^. Additionally, the YT521-B homology (YTH) domain-containing RNA-binding proteins in plants were described as having a responsive function to the oxidative stress caused by the generation of reactive oxygen species (ROS)^51^.

Water deficit affects many aspects of the physiology of plants and particularly their photosynthetic capacity. By identifying SNP photosynthesis-related trait associations and the underlying candidate genes, we presented insights into the genetic basis of those physiological mechanisms. Moreover, the functional categorization of candidate genes corroborated the myriad of metabolic pathways involved in common bean response to water limitation. The Portuguese common bean accessions evaluated here under contrasting water treatments presented a large variability in their photosynthesis-related responses. Some of the accessions with higher pigment contents and better photosynthetic responses were not only related to the Andean or to the Mesoamerican gene pools but also to an intermediate admixture nature between the two original gene pools. Those intermediate accessions might offer complimentary alleles and novel genetic combinations valuable for improving water-deficit tolerance in both gene pools.

One of the main limitations of our study is that we did not measure biomass or yield parameters in our controlled condition experiments. Thus, we could not establish correlations between the leaf and photosynthesis-related traits measured with each accession’s yield, the main driver of any breeding program. However, this preliminary large evaluation was required to select a smaller group of accessions with contrasting photosynthetic responses, for a future more detailed analysis exposing them to different levels of WD, along with plant developmental growth stages, including a recovery period until harvest. The next step will also include analyzing the most promising accessions identified here, under field conditions, using a multilocation design to assess the environmental effect on the analyzed traits through a genotype-by-environment interaction analysis, validating the usefulness of the results obtained in the current controlled study under real-field conditions.

## Materials and methods

### Plant material and growing conditions

One-hundred-and-fifty-eight Portuguese common bean accessions, previously characterized at the molecular level^25^, were used in this study (Supplementary Table S1). This collection includes accessions of Andean, Mesoamerican, and admixed origin. Two Mesoamerican CIAT lines (International Centre for Tropical Agriculture, Cali, Colombia), SER16, and Tio Canela-75, were also included as international references. SER16 is an elite line with superior drought tolerance and Tio Canela-75, a drought-susceptible cultivar^12,52^.

Ten seeds per accession were sown, one seed per pot, in a growth chamber at 26 ± 2 °C during the day and 18 ± 2 °C at night, under a photoperiod of 16 h of light (~295 μmol.m^−2^ s^−1^), with a relative humidity of 50% and a CO_2_ concentration of 370 ppm, approximately. Sowing was done in 8 × 8 × 9-cm plastic pots (0.5 L), filled with Montemor soil/peat/vermiculite [2:1:1 (v/v)], watered to full capacity (100%), and weighted. Three extra pots were filled with the same mixture and put in an oven at 80 °C for 1 week to estimate the average dry weight of the soil mixture. This dry-weight value was used later for the soil water content calculation of each pot.

### Experimental design

Due to growth chamber space constraints, an incomplete block design was applied, with four blocks under the same conditions. In each block, 32–48 accessions were evaluated (ten plants per accession, five well-watered, five under water deficit, the same growth chamber). Eight accessions were occasionally and randomly repeated within blocks.

### Water-deficit imposition

The plants were watered every other day to maintain well-watered conditions until the second trifoliate leaf full expansion. At this point, water deficit (WD) was imposed on five plants per accession by withholding watering until the pot soil-field capacity (FC) reached 40%, with daily FC monitoring by pot weighing. The remaining five plants per accession were kept under well-watered (WW) conditions.

### Photosynthetic performance

When the pot soil water content under WD reached 40% of FC, gas-exchange photosynthetic parameters—stomatal CO_2_ conductance (gs), net CO_2_ assimilation (A), transpiration rate (E), and substomatal CO_2_ concentration (C*i*)—were measured in the youngest nondetached and fully expanded trifoliate leaf. Well-watered plants from the same accessions at the identical developmental stage were evaluated for the same parameters, on the same day. Measurements were carried out using a portable Infra-Red Gas Analyzer system (IRGA, LCpro+ ADC BioScientific Ltd., Hertfordshire, UK), with controlled atmosphere (~370 μmol mol^−1^ CO_2_, 23 ± 2 °C, and 50–60% relative humidity) and a saturating external light source of 1044 μmol m^−2^ s^−1^. A, E, and gs values were used to calculate instantaneous and intrinsic water-use efficiencies (WUE = A/E and WUE_i_ = A/gs, respectively).

### Leaf photosynthetic pigments

Leaf photosynthetic pigments—chlorophylls *a* (C*a*) and *b* (C*b*), and carotenes and xanthophylls (C*cx*)—were quantified using a spectrophotometer according to Wintermans and De Mots^53^ on two leaf disks sampled from the same trifoliate leaf previously used for the IRGA measurements. The sum of C*a* and C*b*, their ratio, and the ratio between the sum of chlorophylls and carotenes and xanthophylls [(C*a* + C*b*)/C*cx*] were calculated. Pigment concentrations were expressed per dry- mass unit.

### Leaf water status and the related morphological parameters

Leaf relative water content (RWC) was calculated to assess the water status of the leaves at the time of the gas-exchange measurements, following a protocol adapted from Čatský^54^, based on the fresh weight (FW), turgid weight (TW), and dry-weight calculation (DW) on three disks per plant sample. Leaf RWC was obtained as RWC (%) = [(FW − DW)/(TW − DW)] × 100. The fresh:dry weight (FW/DW) ratio, as an index of cell water content, was also calculated.

The specific leaf area (SLA, the ratio of leaf-disk area to leaf-disk dry mass) and leaf thickness (LT, the ratio of leaf-disk fresh weight to leaf-disk area) were also calculated.

### Phenotypic data analysis

#### Quality control, variance components, and trait heritability

The phenotypic data acquired were subjected to quality control of residuals, assessing normal distribution through graphical inspection (Q–Q plot), the existence of outliers, and homogeneity of variance. A square-root transformation was applied when needed so that the residuals more closely meet the normality assumptions.

A linear mixed model was fitted per trait as *trait* = *block* + *treatment* + *origin* + *accession* + *treatment* × *origin* + *treatment* × *accession* using the restricted maximum likelihood (REML) variance component analysis framework of Genstat software^55^. Accession, water treatment, the gene pool of the origin of accessions, treatment × accession, and water treatment × gene pool of origin interaction were fitted as fixed, and block as random effects. A Wald test for the significance of fixed effects was performed. Only the Portuguese common bean accessions were included in the statistical model. Tukey’s multiple-comparison tests were applied for mean comparison of 16 photosynthesis-related traits measured among the accession gene pool of origin for each water treatment.

Phenotypic data obtained under WW and WD were analyzed separately. REML was used to fit a mixed model per trait as *trait* = *block* + *accession* + *error*. This model, with accession and block fixed, was applied to estimate the best linear-unbiased estimates (BLUEs) for each accession, used as input for the GWAS. Phenotypic correlations of BLUEs were calculated between traits, followed by a principal component analysis (PCA).

With our original experimental setup, the variance components for accession and error were not correctly estimated. So, a complementary experiment was performed in the same conditions, using ten accessions and five replications, following an alfa-lattice design. This new experiment was combined with the original experiment in the estimation of variance components for accession and error. Broad-sense heritabilities were calculated using vheritability procedure in Genstat software, based on the same model above, but with block and accession as random, and using the combined experiments.

### Genotypic data

#### Association-mapping analysis

Genome-wide association studies were conducted using the QTL library procedures from Genstat software. Only the nonderived photosynthesis-related traits with comparable error variances (A, E, gs, C*a*, C*b*, and C*cx*) were used for the analysis. Adjusted means (BLUEs) of those traits were tested for association with 9,825 SNP markers using the 144 common bean accessions obtained using the Illumina Infinium BARCBean6K_3 BeadChip^TM^ assay and DArTseq^TM^ analysis, and retrieved from a previous study after quality control^56^. SNP markers and accessions with >25% missing data were removed, as well as SNPs with a minor-allele frequency <0.01. SNPs called as heterozygous were set as missing data.

GWAS was performed separately for WW and WD in the mixed-model framework of Genstat software, using kinship matrix with coefficients of co-ancestry between accessions, and fitting markers as fixed and accessions as random terms on REML. Using a threshold level of –log_10_ (*P* value) = 3, the significant marker-trait associations were highlighted. This threshold was set to discard the background noise observed in the Manhattan plots without compromising the identification of potentially interesting regions, which would be missed, for instance, by the too stringent and conservative Bonferroni-corrected threshold of significance. For every SNP significantly associated with a trait, the effect of the minor-frequency SNP variant was calculated.

### Candidate gene identification

After GWAS, a gene was considered a putative candidate for the phenotypic trait under analysis if it contained an associated SNP or was in linkage disequilibrium (LD) with an SNP associated with the trait. LD was previously calculated for each common bean chromosome using the squared coefficient of the correlation between marker pairs, *r*^2^ (ref. ^56^). To consider the existence of adjacent SNP markers in LD with the ones associated with the traits, the *r*^2^ of the neighboring SNPs was investigated using a strict LD-decay threshold (*r*^2^ > 0.2). An LD block or genomic region was then defined to search for putative candidate genes using the *JBrowse* tool in the *Phaseolus vulgaris* v2.1, available at the Phytozome v12 portal (DOE-JGI and USDA-NIFA, http://phytozome.jgi.doe.gov/). The annotation of the candidate genes was obtained from the file “Pvulgaris_442_v2.1.annotation_info.txt”, available in the same portal, and *KEGG*/*KOG*/*PFAM*/*PANTHER*/*Gene Ontology (GO)* database identifiers were used to make inferences about the pathways involved and possible roles of candidate genes on the analyzed traits.

Candidate gene’s functional characterization was obtained using the MapMan web tools and Mercator4 v2.0 (https://www.plabipd.de/portal/mercator4)^57^. Cytoscape software^58^, version 3.8.0, was used to visualize the molecular-interaction networks associated with each trait.

## Supplementary information

Supplementary Figure S1

Supplementary Figures S2-S4

Supplementary Figures S5

Supplementary Figures S6

Supplementary_Table S1

Supplementary Table S2

Supplementary Table S3

Supplementary Table S4

Supplementary File S5

Supplementary File S6

Supplementary Table S7

Supplementary Table S8
