# Supplementary material for: Common bean SNP alleles and candidate genes affecting photosynthesis under contrasting water regimes
Source: Hortic Res. 2021 Jan 1;8:4. doi: 10.1038/s41438-020-00434-6 (PMC7775448; doi:10.1038/s41438-020-00434-6)
Supplement: Supplementary file 1 — Supplementary Figure S1 [file 41438_2020_434_MOESM1_ESM.pdf]

Figure S1: Histograms of the BLUEs for the 16 traits evaluated in 160 common bean accessions and measured under well-watered (WW, in blue) and water-deficit (WD, in grey) conditions. The Mesoamerican reference lines SER16 and Tio Canela-75 are represented by a black and an orange arrow, respectively.

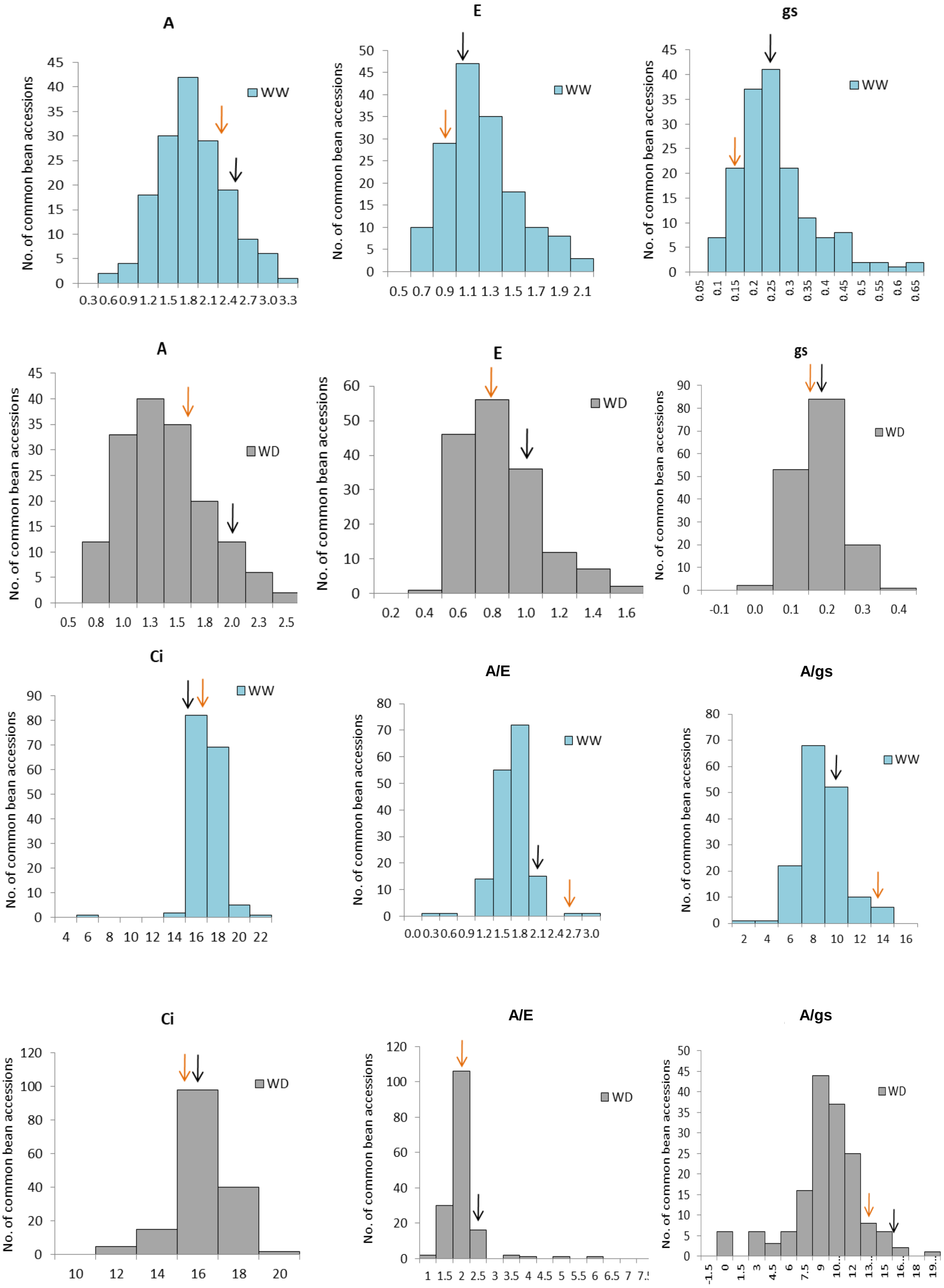

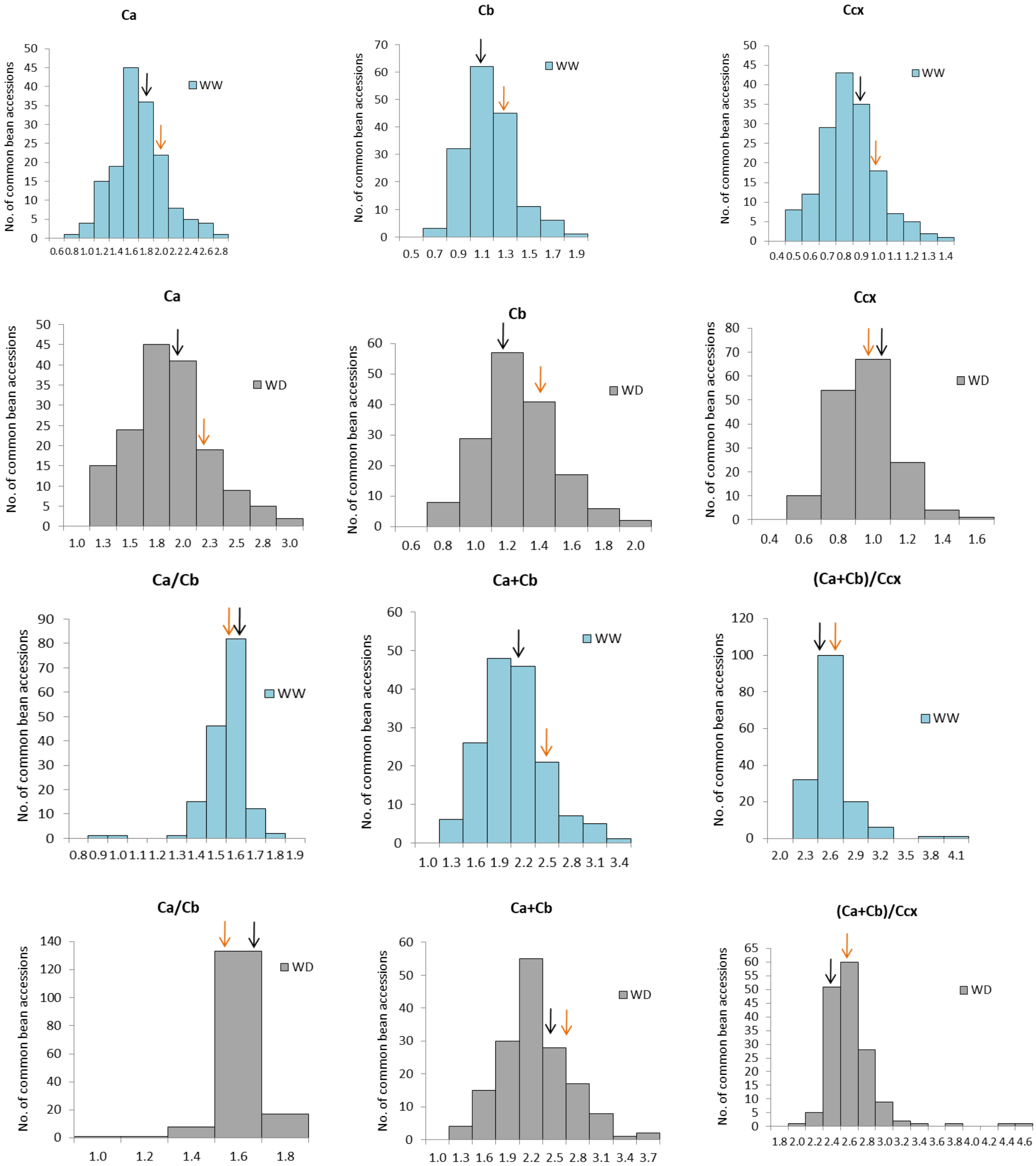
Ca
No. of common bean accessions
WW
0.6 0.8 1.0 1.2 1.4 1.6 1.8 2.0 2.2 2.4 2.6 2.8
Cb
No. of common bean accessions
WW
0.5 0.7 0.9 1.1 1.3 1.5 1.7 1.9
Ccx
No. of common bean accessions
WW
0.4 0.5 0.6 0.7 0.8 0.9 1.0 1.1 1.2 1.3 1.4
Ca
No. of common bean accessions
WD
1.0 1.3 1.5 1.8 2.0 2.3 2.5 2.8 3.0
Cb
No. of common bean accessions
WD
0.6 0.8 1.0 1.2 1.4 1.6 1.8 2.0
Ccx
No. of common bean accessions
WD
0.4 0.6 0.8 1.0 1.2 1.4 1.6
Ca/Cb
No. of common bean accessions
WW
0.8 0.9 1.0 1.1 1.2 1.3 1.4 1.5 1.6 1.7 1.8 1.9
Ca+Cb
No. of common bean accessions
WW
1.0 1.3 1.6 1.9 2.2 2.5 2.8 3.1 3.4
(Ca+Cb)/Ccx
No. of common bean accessions
WW
2.0 2.3 2.6 2.9 3.2 3.5 3.8 4.1
Ca/Cb
No. of common bean accessions
WD
1.0 1.2 1.4 1.6 1.8
Ca+Cb
No. of common bean accessions
WD
1.0 1.3 1.6 1.9 2.2 2.5 2.8 3.1 3.4 3.7
(Ca+Cb)/Ccx
No. of common bean accessions
WD
1.8 2.0 2.2 2.4 2.6 2.8 3.0 3.2 3.4 3.6 3.8 4.0 4.2 4.4 4.6

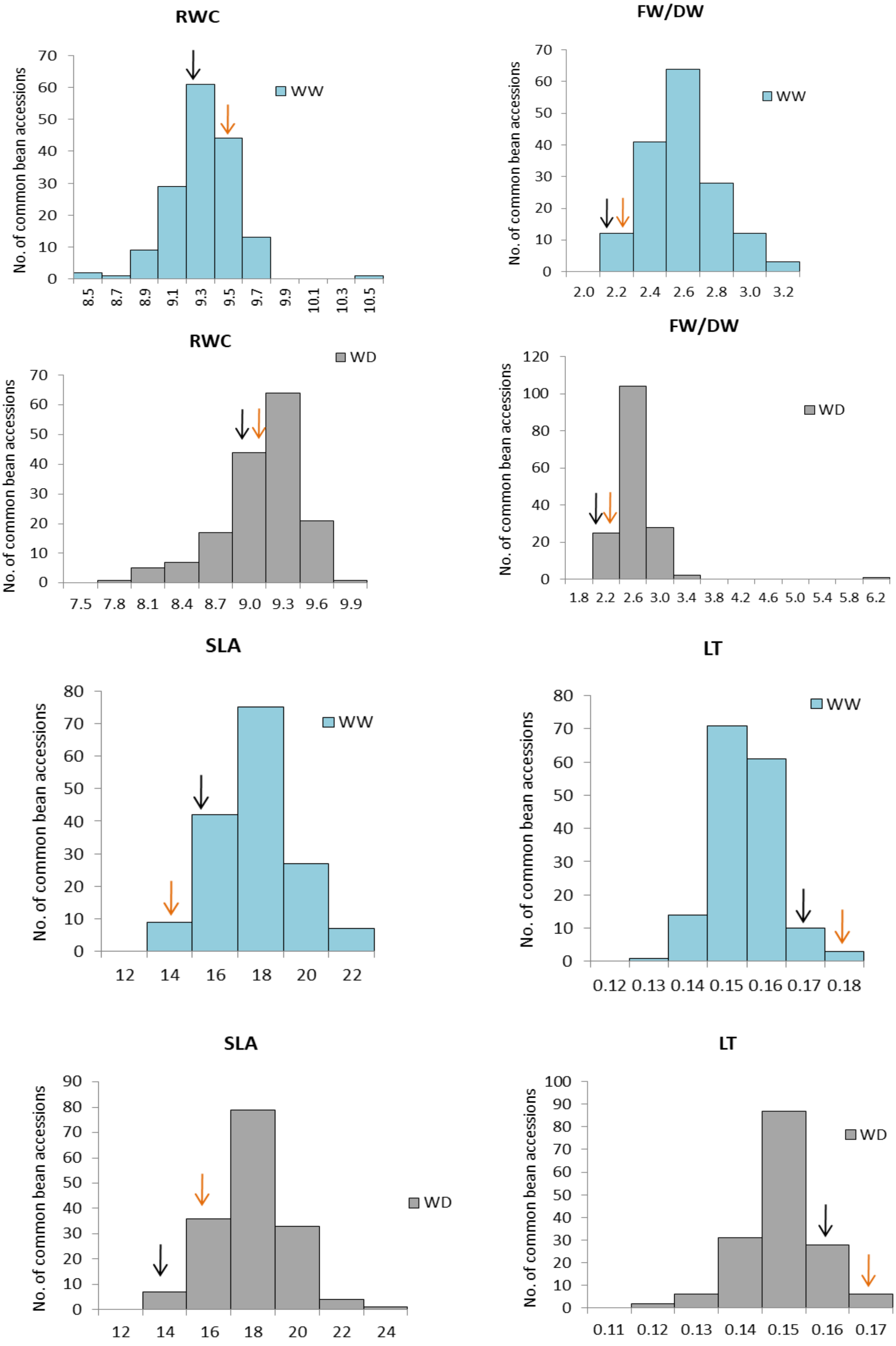
RWC
WW
No. of common bean accessions
70
60
50
40
30
20
10
0
8.5
8.7
8.9
9.1
9.3
9.5
9.7
9.9
10.1
10.3
10.5
FW/DW
WW
No. of common bean accessions
70
60
50
40
30
20
10
0
2.0
2.2
2.4
2.6
2.8
3.0
3.2
RWC
WD
No. of common bean accessions
70
60
50
40
30
20
10
0
7.5
7.8
8.1
8.4
8.7
9.0
9.3
9.6
9.9
FW/DW
WD
No. of common bean accessions
120
100
80
60
40
20
0
1.8
2.2
2.6
3.0
3.4
3.8
4.2
4.6
5.0
5.4
5.8
6.2
SLA
WW
No. of common bean accessions
80
70
60
50
40
30
20
10
0
12
14
16
18
20
22
LT
WW
No. of common bean accessions
80
70
60
50
40
30
20
10
0
0.12
0.13
0.14
0.15
0.16
0.17
0.18
SLA
WD
No. of common bean accessions
90
80
70
60
50
40
30
20
10
0
12
14
16
18
20
22
24
LT
WD
No. of common bean accessions
100
90
80
70
60
50
40
30
20
10
0
0.11
0.12
0.13
0.14
0.15
0.16
0.17
