# Supplementary material for: Common bean SNP alleles and candidate genes affecting photosynthesis under contrasting water regimes
Source: Hortic Res. 2021 Jan 1;8:4. doi: 10.1038/s41438-020-00434-6 (PMC7775448; doi:10.1038/s41438-020-00434-6)
Supplement: Supplementary file 2 — Supplementary Figures S2-S4 [file 41438_2020_434_MOESM2_ESM.pdf]

Figures S2 to S4

For all the plots, the y-axis represents the $-\log_{10}$ (P-value) of 9,825 SNPs, and the x-axis shows their chromosomal positions across the common bean genome. The horizontal red line indicates the significance threshold ($P = 1\times10^{-3}$).

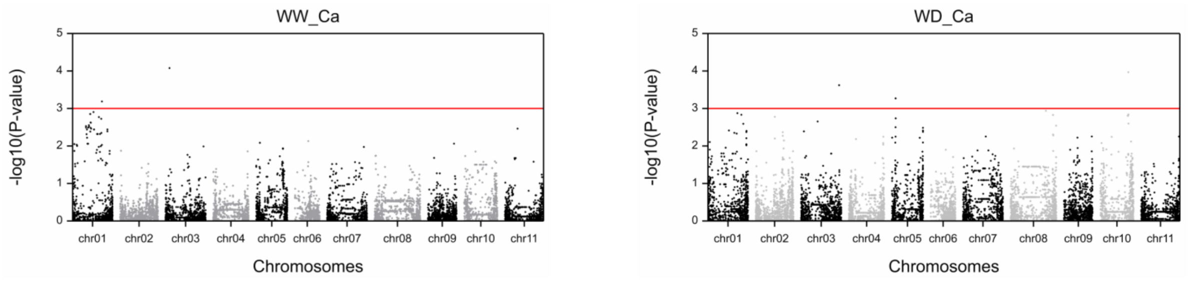

Figure S2: Manhattan plot depicting the genome-wide association results for chlorophyll *a* concentration (C*a*) in 144 Portuguese common bean accessions, under well-watered (WW) and water deficit (WD) conditions.

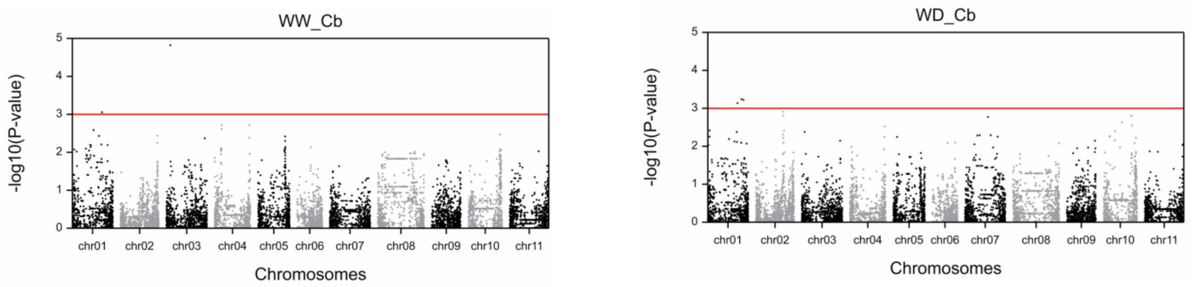

Figure S3: Manhattan plot depicting the genome-wide association results for chlorophyll *b* concentration (C*b*) in 144 Portuguese common bean accessions, under well-watered (WW) and water deficit (WD) conditions.

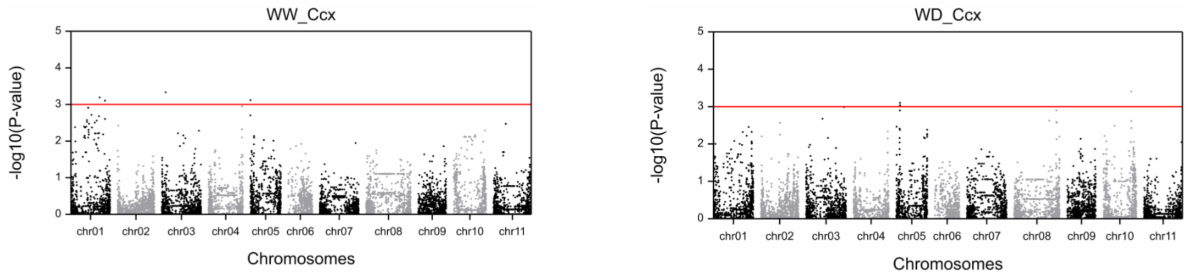

Figure S4: Manhattan plot depicting the genome-wide association results for carotenes and xanthophylls concentration (C*cx*) in 144 Portuguese common bean accessions, under well-watered (WW) and water deficit (WD) conditions
