# Supplementary material for: Common bean SNP alleles and candidate genes affecting photosynthesis under contrasting water regimes
Source: Hortic Res. 2021 Jan 1;8:4. doi: 10.1038/s41438-020-00434-6 (PMC7775448; doi:10.1038/s41438-020-00434-6)

Figure S5: Frequency of the variant allele of the 75 SNPs associated with six photosynthesis-related traits (A, E, gs, Ca, Cb, Ccx) according to the gene pool of origin of the Portuguese common bean accessions. The ten SNPs highlighted with an asterisk (*) are those having a variant allele with a negative effect on the trait. The SNPs are represented according to their chromosomal order.

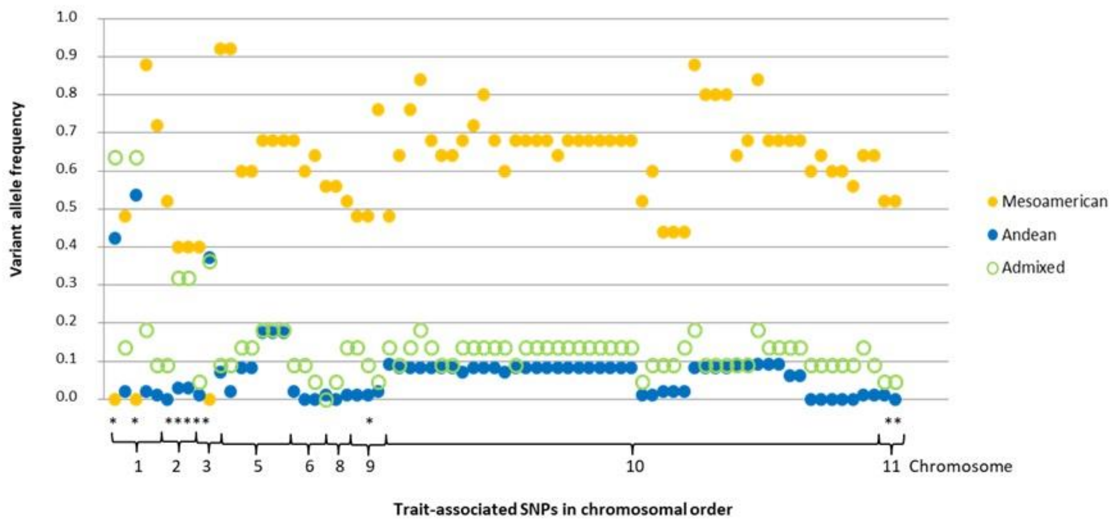

Supplement: Supplementary file 3 — Supplementary Figures S5 [file 41438_2020_434_MOESM3_ESM.pdf]
