# Supplementary material for: Common bean SNP alleles and candidate genes affecting photosynthesis under contrasting water regimes
Source: Hortic Res. 2021 Jan 1;8:4. doi: 10.1038/s41438-020-00434-6 (PMC7775448; doi:10.1038/s41438-020-00434-6)

Figure S6: Network analysis of the candidate genes identified under WW and WD for A, E, gs, C*a*, and C*cx*, using the MapMan functional categories. The circles represent the genes, and green diamonds the traits. The candidate genes in blue circles contained or were in LD with the strongest associated SNPs.

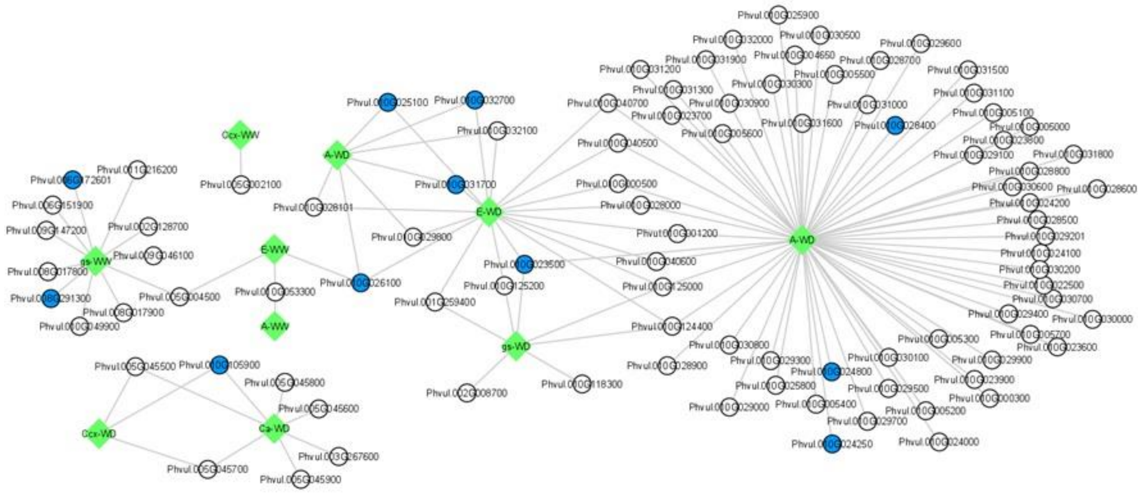

Supplement: Supplementary file 4 — Supplementary Figures S6 [file 41438_2020_434_MOESM4_ESM.pdf]
