# Supplementary material for: Common bean SNP alleles and candidate genes affecting photosynthesis under contrasting water regimes
Source: Hortic Res. 2021 Jan 1;8:4. doi: 10.1038/s41438-020-00434-6 (PMC7775448; doi:10.1038/s41438-020-00434-6)
Supplement: Supplementary file 5 — Supplementary_Table S1 [file 41438_2020_434_MOESM5_ESM.pdf]

Table S1: Passport information, including geographic origin, of the Portuguese common bean collection used in this study. The result from the structure analysis previous done (Leitão et al. 2017) is included.

Accession	Region of origin (Portugal)	District	Latitude	Longitude	Altitude (m)	Structure_K3Group[1]	
g0579	northern interior	Bragança	41°09'0.000"N	6°48'0.000"W	460	B1M	Mixed origin
g0583	northern interior	Bragança	41°09'0.000"N	6°48'0.000"W	460	B1M	Mixed origin
g0584	northern interior	Bragança	41°09'0.000"N	6°48'0.000"W	460	AM	Mixed origin
g0587	northern interior	Bragança	41°32'0.000"N	6°57'0.000"W	570	B1	Andean
g0592	northern interior	Bragança	41°32'0.000"N	6°57'0.000"W	570	B2	Andean
g0600	northern interior	Bragança	41°20'0.000"N	6°43'0.000"W	749	B2M	Mixed origin
g0601	northern interior	Bragança	41°20'0.000"N	6°43'0.000"W	749	B1	Andean
g0602	northern interior	Bragança	41°32'0.000"N	6°57'0.000"W	570	AM	Mixed origin
g0620	northern interior	Bragança	41°32'0.000"N	6°57'0.000"W	570	B2	Andean
g0621	northern interior	Bragança	41°32'0.000"N	6°57'0.000"W	570	B1	Andean
g0623	northern interior	Bragança	41°32'0.000"N	6°57'0.000"W	n/a	A	Mesoamerican
g0632	northern interior	Bragança	41°20'0.000"N	6°43'0.000"W	749	B2	Andean
g0633	northern interior	Bragança	41°20'0.000"N	6°43'0.000"W	749	B2	Andean
g0635	northern interior	Bragança	41°20'0.000"N	6°43'0.000"W	749	B2	Andean
g0638	n/a	n/a	n/a	n/a	n/a	n/a	n/a
g0639	northern interior	Bragança	41°48'0.000"N	6°45'0.000"W	673	B2M	Mixed origin
g0642	northern interior	Bragança	41°29'0.000"N	6°16'0.000"W	679	B2	Andean
g0644	northern interior	Bragança	41°29'0.000"N	6°16'0.000"W	679	B2	Andean
g0645	northern interior	Bragança	41°29'0.000"N	6°16'0.000"W	679	B2	Andean
g0648	northern interior	Bragança	41°29'0.000"N	6°16'0.000"W	679	B2	Andean
g0654	northern interior	Bragança	41°48'0.000"N	6°45'0.000"W	673	A	Mesoamerican
g0667	northern interior	Bragança	41°48'0.000"N	6°45'0.000"W	673	B2	Andean
g0670	northern interior	Bragança	41°48'0.000"N	6°45'0.000"W	673	B2	Andean
g0671	northern interior	Bragança	41°48'0.000"N	6°45'0.000"W	673	B2	Andean
g0675	northern interior	Bragança	41°48'0.000"N	6°45'0.000"W	673	B2M	Mixed origin
g0677	n/a	n/a	n/a	n/a	n/a	n/a	n/a
g0695	interior North	Bragança	41°48'0.000"N	6°45'0.000"W	673	B2	Andean
g0698	northern interior	Bragança	41°45'0.000"N	6°30'0.000"W	700	B1	Andean
g0700	northern interior	Bragança	41°45'0.000"N	6°30'0.000"W	700	B2	Andean
g0706	central north	Vila Real	41°44'0.000"N	7°28'0.000"W	368	B2	Andean
g0735	northern interior	Bragança	41°50'8.000"N	7°00'0.500"W	687	B2	Andean
g0736	northern interior	Bragança	41°50'8.000"N	7°00'0.500"W	687	B2	Andean
g0737	central north	Vila Real	41°49'0.000"N	7°47'0.000"W	990	B2	Andean
g0747	interior North	Bragança	41°29'2.400"N	7°10'3.900"W	218	B2	Andean
g0748	northern interior	Bragança	41°29'2.400"N	7°10'3.900"W	218	A	Mesoamerican
g1631	central south	Lisboa	38°47'0.000"N	9°23'0.000"W	180	B2	Andean
g1636	central south	Lisboa	38°56'0.000"N	9°19'0.000"W	242	A	Mesoamerican
g1644	central south	Lisboa	39°49'0.000"N	9°10'0.000"W	21	A	Mesoamerican
g1651	central south	Lisboa	38°56'0.000"N	9°19'0.000"W	242	A	Mesoamerican
g1653	central south	Lisboa	38°56'0.000"N	9°19'0.000"W	242	B2	Andean
g1654	central south	Lisboa	38°56'0.000"N	9°19'0.000"W	242	B1	Andean

g1662	central south	Lisboa	38°56'0.000"N	9°19'0.000"W	242	B2	Andean
g1663	central south	Lisboa	38°56'0.000"N	9°19'0.000"W	242	B1	Andean
g1867	northern interior	Bragança	41°29'2.400"N	7°10'3.900"W	218	A	Mesoamerican
g1871	northern interior	Bragança	41°29'2.400"N	7°10'3.900"W	218	B2	Andean
g1877	northern interior	Bragança	41°29'2.400"N	7°10'3.900"W	218	B2	Andean
g1883	northern interior	Bragança	41°29'2.400"N	7°10'3.900"W	218	B1	Andean
g1889	northern interior	Bragança	41°32'0.000"N	6°57'0.000"W	570	B2	Andean
g1892	northern interior	Bragança	41°32'0.000"N	6°57'0.000"W	570	A	Mesoamerican
g1893	northern interior	Bragança	41°32'0.000"N	6°57'0.000"W	n/a	B2	Andean
g1897	interior North	Bragança	41°20'0.000"N	6°43'0.000"W	749	A	Mesoamerican
g1911	northern interior	Bragança	41°29'0.000"N	6°16'0.000"W	679	B2	Andean
g1917	northern interior	Bragança	41°29'0.000"N	6°16'0.000"W	679	B1	Andean
g1918	northern interior	Bragança	41°48'0.000"N	6°45'0.000"W	673	A	Mesoamerican
g1926	northern interior	Bragança	41°48'0.000"N	6°45'0.000"W	673	B2	Andean
g1927	interior North	Bragança	41°48'0.000"N	6°45'0.000"W	673	B2	Andean
g1932	northern interior	Bragança	41°48'0.000"N	6°45'0.000"W	673	A	Mesoamerican
g1933	northern interior	Bragança	41°48'0.000"N	6°45'0.000"W	673	B2	Andean
g1937	northern interior	Bragança	41°48'0.000"N	6°45'0.000"W	673	B1	Andean
g1938	northern interior	Bragança	41°48'0.000"N	6°45'0.000"W	673	A	Mesoamerican
g1943	northern interior	Bragança	41°48'0.000"N	6°45'0.000"W	673	B1	Andean
g1944	northern interior	Bragança	41°48'0.000"N	6°45'0.000"W	673	B2	Andean
g1948	northern interior	Bragança	41°48'0.000"N	6°45'0.000"W	673	AM	Mixed origin
g1952	northern interior	Bragança	41°48'0.000"N	6°45'0.000"W	673	A	Mesoamerican
g1955	northern interior	Bragança	41°32'0.000"N	6°57'0.000"W	570	A	Mesoamerican
g1956	northern interior	Bragança	41°32'0.000"N	6°57'0.000"W	570	B2M	Mixed origin
g1961	northern interior	Bragança	41°32'0.000"N	6°57'0.000"W	570	B2	Andean
g1964	northern interior	Bragança	41°32'0.000"N	6°57'0.000"W	570	A	Mesoamerican
g1966	central north	Vila Real	41°44'0.000"N	7°28'0.000"W	368	B2	Andean
g1975	central north	Vila Real	41°44'0.000"N	7°28'0.000"W	368	B2	Andean
g1976	central north	Vila Real	41°36'0.000"N	07°18'0.000"W	425	B2	Andean
g1979	central north	Vila Real	41°36'0.000"N	07°18'0.000"W	425	A	Mesoamerican
g1984	central north	Vila Real	41°36'0.000"N	07°18'0.000"W	425	B2	Andean
g2081	Madeira	Funchal	32°40'0.000"N	17°04'0.000"W	50	B2	Andean
g2105	Madeira	Funchal	n/a	n/a	n/a	B2	Andean
g2126	Madeira	Funchal	32°45'0.000"N	16°49'0.000"W	250	B2	Andean
g2155	Madeira	Funchal	32°47'0.000"N	17°02'0.000"W	150	B2	Andean
g2159	Madeira	Funchal	32°49'0.000"N	17°06'0.000"W	150	B1M	Mixed origin
g2179	Madeira	Funchal	32°43'0.000"N	16°57'0.000"W	690	A	Mesoamerican
g2189	Madeira	Funchal	32°43'0.000"N	17°01'0.000"W	500	B1	Andean
g2192	Madeira	Funchal	32°43'0.000"N	17°01'0.000"W	500	B2	Andean
g4038	central north	Viseu	40°53'7.600"N	07°42'5.000"W	862	B1	Andean
g4044	central north	Viseu	40°53'9.700"N	07°43'3.900"W	844	B2	Andean
g4048	central north	Guarda	40°53'0.100"N	07°48'4.000"W	774	B2	Andean
g4049	central north	Guarda	40°53'0.100"N	07°48'4.000"W	774	B2	Andean
g4050	central north	Guarda	40°53'0.100"N	07°48'4.000"W	774	B2	Andean
g4051	central north	Guarda	40°53'0.100"N	07°48'4.000"W	774	B1	Andean

g4064	n/a	n/a	n/a	n/a	n/a	n/a	n/a
g4067	central north	Viseu	40°50'5.500"N	07°56'2.400"W	471	B1	Andean
g4070	central north	Viseu	40°54'8.300"N	07°58'3.800"W	502	B1	Andean
g4071	central north	Viseu	40°54'8.300"N	07°58'3.800"W	502	B1	Andean
g4072	central north	Viseu	40°54'8.300"N	07°58'3.800"W	502	B2	Andean
g4073	central north	Viseu	n/a	n/a	n/a	A	Mesoamerican
g4074	n/a	n/a	n/a	n/a	n/a	n/a	n/a
g4081	central north	Viseu	40°57'1.300"N	07°54'8.400"W	867	B1M	Mixed origin
g4085	central north	Viseu	40°41'8.100"N	08°04'9.700"W	567	B1M	Mixed origin
g4088	central north	Viseu	40°41'8.100"N	08°04'9.700"W	567	B1	Andean
g4097	central north	Viseu	40°41'8.100"N	08°04'9.700"W	567	B1	Andean
g4099	n/a	n/a	n/a	n/a	n/a	n/a	n/a
g4100	central north	Viseu	40°39'3.300"N	08°09'1.800"W	747	B1	Andean
g4108	central north	Viseu	40°38'0.400"N	08°03'1.100"W	434	B1	Andean
g4110	central north	Viseu	40°39'0.000"N	07°54'0.100"W	475	B1M	Mixed origin
g4119	central north	Viseu	40°39'0.000"N	07°54'0.100"W	475	B1	Andean
g4120	central north	Viseu	40°39'0.000"N	07°54'0.100"W	475	B1	Andean
g4127	central north	Guarda	40°45'7.500"N	07°34'4.400"W	609	B2	Andean
g4133	central north	Guarda	40°45'0.500"N	07°32'1.700"W	544	B2	Andean
g4144	central north	Guarda	40°51'3.200"N	007°30'1.900"W	618	A	Mesoamerican
g4149	central north	Guarda	40°19'5.100"N	007°41'1.800"W	794	B1	Andean
g4150	central north	Guarda	40°19'5.100"N	007°41'1.800"W	794	B1	Andean
g4162	central north	Guarda	40°31'5.300"N	007°34'2.300"W	459	B2M	Mixed origin
g4164	central north	Guarda	40°31'5.300"N	007°34'2.300"W	459	B1	Andean
g4179	central north	Guarda	40°39'0.20"N	07°24'5.300"W	441	B2	Andean
g4182	central north	Guarda	40°40'1.200"N	07°24'7.000"W	426	A	Mesoamerican
g4185	central north	Coimbra	40°19'9.200"N	07°50'5.500"W	269	B1	Andean
g4189	central north	Coimbra	40°19'9.200"N	07°50'5.500"W	269	B2	Andean
g4195	n/a	n/a	n/a	n/a	n/a	n/a	n/a
g4295	south	Faro	37°18'0.000"N	08°48'0.000"W	36	B1	Andean
g4300	south	Faro	37°00'0.000"N	07°56'0.000"W	9	B2	Andean
g4306	south	Faro	n/a	n/a	n/a	B1	Andean
g5249	central north	Viseu	40°53'1.920"N	08°05'9.830"	453	A	Mesoamerican
g5285	north coast	Braga	41°30'0.00"N	07°59'0.000"W	300	B1	Andean
g5286	north coast	Braga	41°30'0.00"N	07°59'0.000"W	300	A	Mesoamerican
g5287	north coast	Braga	41°30'0.00"N	07°59'0.000"W	300	A	Mesoamerican
g5288	central north	Aveiro	40°38'0.00"N	08°39'0.000"W	8	B2	Andean
g5289	n/a	n/a	n/a	n/a	n/a	n/a	n/a
g5291	South	Faro	37°00'0.000"N	07°56'0.000"W	9	B1	Andean
g5292	South	Faro	37°00'0.000"N	07°56'0.000"W	9	A	Mesoamerican
g5295	South	Faro	37°08'0.00"N	08°01'0.000"W	171	B2	Andean
g5296	South	Faro	37°18'0.000"N	08°48'0.000"W	36	B2M	Mixed origin
g5297	South	Faro	37°08'0.00"N	08°01'0.000"W	171	B2M	Mixed origin
g5298	northern interior	Bragança	41°09'0.000"N	6°48'0.000"W	460	B1	Andean
g5300	northern interior	Bragança	41°48'0.000"N	6°45'0.000"W	673	B2	Andean
g5306	n/a	n/a	n/a	n/a	n/a	n/a	n/a

g5363	central south	Lisboa	38°56'0.000"N	9°19'0.000"W	242	B2	Andean
g5366	central north	Guarda	40°32'0.000"N	07°16'0.000"W	540	B2	Andean
g5367	central north	Guarda	40°32'0.000"N	07°16'0.000"W	540	B2	Andean
g5368	central north	Guarda	40°32'0.000"N	07°16'0.000"W	540	B1M	Mixed origin
g5369	central north	Aveiro	40°38'0.00"N	08°39'0.000"W	8	B1	Andean
g5370	north coast	Braga	41°32'0.000"N	08°36'0.000"W	34	A	Mesoamerican
g5371	north coast	Braga	41°32'0.000"N	08°36'0.000"W	34	B2	Andean
g5372	central south	Leiria	39°21'0.00"N	09°09'0.000"W	51	B1	Andean
g5375	n/a	n/a	n/a	n/a	n/a	n/a	n/a
g5376	south	Faro	37°00'0.000"N	07°56'0.000"W	9	B2M	Mixed origin
g5377	south	Faro	37°00'0.000"N	07°56'0.000"W	9	B2	Andean
g5378	central south	Oeste	38°56'0.000"N	9°19'0.000"W	240	B1M	Mixed origin
g5379	northern interior	Bragança	41°48'0.000"N	6°45'0.000"W	673	B2M	Mixed origin
g5380	north coast	Braga	41°32'0.000"N	08°36'0.000"W	34	B2	Andean
g5381	north coast	Braga	41°32'0.000"N	08°36'0.000"W	34	B2	Andean
g5382	north coast	Braga	n/a	n/a	n/a	B1	Andean
g5383	north coast	Braga	n/a	n/a	n/a	B1	Andean
g5384	central south	Santarém	n/a	n/a	n/a	B1	Andean
g5385	central north	Guarda	n/a	n/a	n/a	B2	Andean
g5386	central north	Guarda	n/a	n/a	n/a	B1	Andean
g5387	central north	Guarda	n/a	n/a	n/a	B1M	Mixed origin
g5388	central north	Viseu	n/a	n/a	n/a	B1M	Mixed origin
g5389	central north	Viseu	n/a	n/a	n/a	B1	Andean
g5391	n/a	n/a	n/a	n/a	n/a	n/a	n/a
gTarrestre	north coast	Braga	n/a	n/a	n/a	A	Mesoamerican

[1]Results from Structure analysis with 21 SSRs and 10 individuals per accession, done together with accessions representative of the original Andean and Mesoamerican gene pools (Leitão *et al*., 2017)

**K3Group**	**Description**	**Note**	
A	Q > 0.75 in Cluster A		Cluster A more related to the
AM	0.50 < Q < 0.75 in Cluster A	Mixed origin	original Mesoamerican gene pool
B1	Q > 0.75 in Cluster B1		
B1M	0.50 < Q < 0.75 in Cluster B1	Mixed origin	
B2	Q > 0.75 in Cluster B2		Clusters B1 and B2 more related to
B2M	0.50 < Q < 0.75 in Cluster B2	Mixed origin	the original Andean gene pool
