# Supplementary material for: Common bean SNP alleles and candidate genes affecting photosynthesis under contrasting water regimes
Source: Hortic Res. 2021 Jan 1;8:4. doi: 10.1038/s41438-020-00434-6 (PMC7775448; doi:10.1038/s41438-020-00434-6)
Supplement: Supplementary file 6 — Supplementary Table S2 [file 41438_2020_434_MOESM6_ESM.docx]

**Table S2:** REML analysis for 16 traits evaluated in 160 common bean accessions (Acc) under two water treatments (Treat): well-watered (WW) and water-deficit (WD) conditions. The statistical model included as fixed factors the accessions, the water treatment, the gene pool of origin of the accessions, and the interactions between treatment and accessions and treatment and origin. Traits mean and range values are shown for the Portuguese (PT) accessions and for the Mesoamerican lines, SER16 and Tio Canela-75.

| Trait | Chi-square distribution for Wald tests | | | | | Trait mean ± standard deviation  (minimum – maximum) | |
| --- | --- | --- | --- | --- | --- | --- | --- |
|  | Acc | Treat | Origin | Treat x Acc | Treat x Origin | WW | WD |
| RWC (%) | 0.248 | <0.001 | 0.019 | 0.867 | 0.349 | PT accessions  85.07 ± 3.87  (80.97 – 89.26) | PT accessions  80.39 ± 6.18  (72.85 – 86.42) |
|  |  |  |  |  |  | SER16:  82.43 ± 5.28  Tio Canela-75:  87.84 ± 2.23 | SER16:  78.87 ± 5.51  Tio Canela-75:  82.97 ± 3.80 |
| FW/DW | <0.001 | 0.190 | 0.109 | <0.001 | 0.004 | PT accessions  6.30 ± 0.82  (5.43 – 7.23) | PT accessions  6.01 ± 0.78  (5.17 – 6.91) |
|  |  |  |  |  |  | SER16:  4.20 ± 0.30  Tio Canela-75:  4.69 ± 0.49 | SER16:  4.44 ± 0.82  Tio Canela-75:  4.93 ± 0.41 |
| A  (µmol CO_2_.m^-2^.s^-1^) | 0.047 | <0.001 | <0.001 | 0.987 | 0.064 | PT accessions  3.319 ± 1.005  (2.296 – 4.476) | PT accessions  1.831 ± 0.667  (1.163 – 2.635) |
|  |  |  |  |  |  | SER16:  5.809 ± 1.609  Tio Canela-75:  5.087 ± 1.602 | SER16:  3.068 ± 1.341  Tio Canela-75:  1.805 ± 0.628 |
| E  (mmol H_2_O.m^-2^.s^-1^) | 0.887 | <0.001 | <0.001 | 0.975 | 0.040 | PT accessions  1.369 ± 0.345  (1.014 - 1.764) | PT accessions  0.618 ± 0.204  (0.416 – 0.861) |
|  |  |  |  |  |  | SER16:  1.456 ± 0.499  Tio Canela-75:  0.823 ± 0.300 | SER16:  0.444 ± 0.281  Tio Canela-75:  0.289 ± 0.082 |
| gs  (mol CO_2_.m^-2^.s^-1^) | 0.847 | <0.001 | <0.001 | 0.896 | <0.001 | PT accessions  0.0683 ± 0.0252  (0.0441 – 0.0984) | PT accessions  0.0201 ± 0.0086  (0.0119 – 0.0301) |
|  |  |  |  |  |  | SER16:  0.0820 ± 0.0390  Tio Canela-75:  0.0367 ± 0.0149 | SER16:  0.0158 ± 0.0117  Tio Canela-75:  0.0080 ± 0.0030 |
| C*i*  (μmol CO_2_.mol*^-^*^1^ air) | 0.035 | <0.001 | 0.738 | 0.979 | 0.189 | PT accessions  256 ± 25  (229 – 286) | PT accessions  235 ± 35  (201 – 275) |
|  |  |  |  |  |  | SER16:  200.1 ± 1.2  Tio Canela-75:  200.7 ± 1.3 | SER16:  202.1 ± 0.8  Tio Canela-75:  201.4 ± 0.8 |
| C*a*  (mg.g^-1^ DW) | 0.074 | <0.001 | 0.004 | 1.000 | 0.753 | PT accessions  2.697 ± 0.730  (1.958 – 3.558) | PT accessions  3.255 ± 1.003  (2.255 – 4.467) |
|  |  |  |  |  |  | SER16:  2.922 ± 0.970  Tio Canela-75:  3.807 ± 1.304 | SER16:  3.853 ± 0.567  Tio Canela-75:  4.318 ± 1.372 |
| C*b*  (mg.g^-1^ DW) | <0.001 | <0.001 | <0.001 | 0.998 | 0.293 | PT accessions  1.185 ± 0.326  (0.858 – 1.573) | PT accessions  1.417 ± 0.437  (0.989 – 1.969) |
|  |  |  |  |  |  | SER16:  1.170 ± 0.263  Tio Canela-75:  1.673 ± 0.544 | SER16:  1.507 ± 0.175  Tio Canela-75:  1.860 ± 0.630 |
| C*cx*  (mg.g^-1^ DW) | <0.001 | 0.001 | 0.001 | 1.000 | 0.330 | PT accessions  0.642 ± 0.163  (0.476 – 0.833) | PT accessions  0.750 ± 0.223  (0.524 – 1.013) |
|  |  |  |  |  |  | SER16:  0.709 ± 0.266  Tio Canela-75:  0.807 ± 0.235 | SER16:  0.895 ± 0.225  Tio Canela-75:  0.876 ± 0.274 |
| SLA  (cm^2^.g^-1^ DW) | <0.001 | 0.039 | 0.319 | 1.000 | 0.635 | PT accessions  287 ± 42  (244 – 337) | PT accessions  297 ± 42  (253 – 347) |
|  |  |  |  |  |  | SER16:  202 ± 20  Tio Canela-75:  193 ± 13 | SER16:  209 ± 15  Tio Canela-75:  214 ± 59 |
| LT (mg FW.cm^-2^) | <0.001 | <0.001 | 0.001 | 1.000 | 0.645 | PT accessions  0.022 ± 0.001  (0.021 – 0.024) | PT accessions  0.021 ± 0.001  (0.019 – 0.022) |
|  |  |  |  |  |  | SER16:  0.026 ± 0.001  Tio Canela-75:  0.029 ± 0.002 | SER16:  0.022 ± 0.002  Tio Canela-75:  0.026 ± 0.002 |
| WUE=A/E | <0.001 | <0.001 | <0.001 | <0.001 | <0.001 | PT accessions  2.43 ± 0.66  (1.78 – 3.18) | PT accessions  4.13 ± 2.08  (2.21 – 6.52) |
|  |  |  |  |  |  | SER16:  4.16 ± 0.53  Tio Canela-75:  6.46 ± 1.91 | SER16:  7.68 ± 1.67  Tio Canela-75:  6.25 ± 1.27 |
| WUE_i_=A/gs | <0.001 | <0.001 | 0.001 | <0.001 | 0.036 | PT accessions  63.05 ± 24.27  (40.22 – 92.50) | PT accessions  91.89 ± 48.70  (48.97 – 149.6) |
|  |  |  |  |  |  | SER16:  82.17 ± 28.04  Tio Canela-75:  146.20 ± 40.98 | SER16:  264.00 ± 148.70  Tio Canela-75:  234.40 ± 44.57 |
| C*a* + C*b*  (mg/g DW) | 0.078 | <0.001 | <0.001 | 1.000 | 0.714 | PT accessions  3.88 ± 1.05  (2.82 – 5.12) | PT accessions  4.67 ± 1.42  (3.26 – 6.39) |
|  |  |  |  |  |  | SER16:  4.09 ± 1.22  Tio Canela-75:  5.48 ± 1.84 | SER16:  5.36 ± 0.71  Tio Canela-75:  6.18 ± 1.99 |
| C*a*/C*b* | 0.006 | 0.372 | 0.581 | 1.000 | 0.963 | PT accessions  2.27 ± 0.16  (2.09 – 2.44) | PT accessions  2.31 ± 0.16  (2.11 – 2.47) |
|  |  |  |  |  |  | SER16:  2.44 ± 0.38  Tio Canela-75:  2.27 ± 0.12 | SER16:  2.56 ± 0.26  Tio Canela-75:  2.34 ± 0.13 |
| (C*a*+C*b*)/C*cx* | 1.000 | 0.387 | 0.999 | 1.000 | 0.837 | PT accessions  6.18 ± 0.90  (5.33 – 7.22) | PT accessions  6.45 ± 1.12  (5.52 – 8.25) |
|  |  |  |  |  |  | SER16:  5.97 ± 0.71  Tio Canela-75:  6.76 ± 0.73 | SER16:  6.21 ± 1.33  Tio Canela-75:  7.06 ± 0.70 |

Phenotypic traits: A – net CO_2_ assimilation rate, E – transpiration rate, gs – stomatal conductance of CO_2_, C*i* – sub-stomatal CO_2_ concentration, C*a* – chlorophyll *a* concentration, C*b* - chlorophyll *b* concentration, C*cx* – carotenes and xanthophylls concentration, RWC – leaf relative water content, FW/DW – leaf fresh: dry weight ratio, SLA – specific leaf area, LT – leaf thickness, A/E – instantaneous water use efficiency, A/gs – intrinsic water use efficiency.
