# Supplementary material for: Common bean SNP alleles and candidate genes affecting photosynthesis under contrasting water regimes
Source: Hortic Res. 2021 Jan 1;8:4. doi: 10.1038/s41438-020-00434-6 (PMC7775448; doi:10.1038/s41438-020-00434-6)
Supplement: Supplementary file 7 — Supplementary Table S3 [file 41438_2020_434_MOESM7_ESM.docx]

**Table S3:** Tukey’s test for comparison of means of the 16 traits among gene pools of origin of the 160 common bean accessions for under well-watered (WW) and water deficit (WD) condition. The mean values shown are the adjusted means (BLUEs).

| Trait_Treatment | Mean value of the trait for each gene pool of origin of the Portuguese accessions^1^ | | | | |
| --- | --- | --- | --- | --- | --- |
|  | Andean | Mesoamerican | Admixed | *P (F)* | ^2^SE |
| RWC_WW | 9.232 a | 9.165 a | 9.274 a | 0.268 | 0.0579 |
| RWC_WD | 8.990 b | 8.807 a | 8.979 ab | 0.042 | 0.0800 |
|  |  |  |  |  |  |
| FW/DW _WW | 2.483 a | 2.539 a | 2.483 a | 0.506 | 0.0539 |
| FW/DW _WD | 2.492 a | 2.398 a | 2.367 a | 0.212 | 0.0840 |
|  |  |  |  |  |  |
| A_WW | 1.641 a | 1.989 b | 1.576 a | 0.002 | 0.1132 |
| A_WD | 1.227 a | 1.412 a | 1.175 a | 0.054 | 0.0919 |
|  |  |  |  |  |  |
| E_WW | 1.082 a | 1.285 b | 1.039 a | 0.002 | 0.0680 |
| E_WD | 0.7356 a | 0.8341 a | 0.6967 a | 0.066 | 0.0529 |
|  |  |  |  |  |  |
| gs_WW | 0.2222 a | 0.2954 b | 0.2079 a | 0.001 | 0.0300 |
| gs_WD | 0.1221 a | 0.1384 a | 0.1126 a | 0.325 | 0.0149 |
|  |  |  |  |  |  |
| C*i*_WW | 15.93 a | 15.85 a | 16.08 a | 0.844 | 0.3240 |
| C*i*_WD | 15.35 a | 15.18 a | 15.38 a | 0.839 | 0.3200 |
|  |  |  |  |  |  |
| C*a*_WW | 1.606 a | 1.619 a | 1.475 a | 0.218 | 0.0787 |
| C*a*_WD | 1.746 a | 1.820 a | 1.617 a | 0.127 | 0.0829 |
|  |  |  |  |  |  |
| C*b*_WW | 1.074 a | 1.068 a | 0.973 a | 0.095 | 0.0462 |
| C*b*_WD | 1.159 a | 1.204 a | 1.065 a | 0.080 | 0.0519 |
|  |  |  |  |  |  |
| C*cx* _WW | 0.7847 a | 0.8184 a | 0.7118 a | 0.066 | 0.0382 |
| C*cx* _WD | 0.8399 a | 0.8834 a | 0.7776 a | 0.084 | 0.0390 |
|  |  |  |  |  |  |
| SLA_WW | 16.79 a | 16.90 a | 16.57 a | 0.788 | 0.3950 |
| SLA_WD | 17.20 a | 16.69 a | 16.47 a | 0.108 | 0.4000 |
|  |  |  |  |  |  |
| LT_WW | 0.1488 a | 0.1522 a | 0.1508 a | 0.101 | 0.0019 |
| LT_WD | 0.1439 a | 0.1458 a | 0.1455 a | 0.452 | 0.0020 |
|  |  |  |  |  |  |
| A/E_WW | 1.506 a | 1.566 a | 1.499 a | 0.629 | 0.0713 |
| A/E_WD | 1.856 a | 1.689 a | 1.798 a | 0.591 | 0.1807 |
|  |  |  |  |  |  |
| A/gs_WW | 7.880 a | 7.562 a | 7.216 a | 0.323 | 0.4650 |
| A/gs_WD | 8.861 a | 8.154 a | 8.433 a | 0.575 | 0.7840 |
|  |  |  |  |  |  |
| C*a*+C*b*_WW | 1.933 a | 1.943 a | 1.767 a | 0.164 | 0.0897 |
| C*a*+C*b*_WD | 2.097 a | 2.186 a | 1.936 a | 0.099 | 0.0963 |
|  |  |  |  |  |  |
| C*a*/C*b*_WW | 1.497 a | 1.510 a | 1.510 a | 0.771 | 0.0246 |
| C*a*/C*b*_WD | 1.507 a | 1.525 a | 1.523 a | 0.579 | 0.0218 |
|  |  |  |  |  |  |
| (C*a*+C*b*)/C*cx*_WW | 2.483 a | 2.380 a | 2.487 a | 0.161 | 0.0605 |
| (C*a*+C*b*)/C*cx*_WD | 2.525 a | 2.510 a | 2.500 a | 0.930 | 0.0728 |
|  |  |  |  |  |  |

^1^For each line, different letters in Tukey’s test means significantly differences (at 95% confidence interval) in the trait means between gene pools of origin of the Portuguese common bean accessions. ^2^Standard error of differences of means.

Phenotypic traits: A_n_ – net CO_2_ assimilation rate, E – transpiration rate, gs – stomatal conductance of CO_2_, C*i* – sub-stomatal CO_2_ concentration, C*a* – chlorophyll *a* concentration, C*b* - chlorophyll *b* concentration, C*cx* – carotenes and xanthophylls concentration, RWC – leaf relative water content, FW/DW – leaf fresh: dry weight ratio, SLA – specific leaf area, LT – leaf thickness, A_n_/E – instantaneous water use efficiency, A_n_/gs – intrinsic water use efficiency.
