# Supplementary material for: Common bean SNP alleles and candidate genes affecting photosynthesis under contrasting water regimes
Source: Hortic Res. 2021 Jan 1;8:4. doi: 10.1038/s41438-020-00434-6 (PMC7775448; doi:10.1038/s41438-020-00434-6)
Supplement: Supplementary file 8 — Supplementary Table S4 [file 41438_2020_434_MOESM8_ESM.docx]

**Table S4:** Estimates of the heritability and variance components for accession, block and residual of the 16 traits measured in the Portuguese common bean collection under well-watered and water deficit conditions using the mixed model with the accession factor fitted as a random term. WW – well-water, WD – water deficit

| Trait – Water treatment | Broad-sense heritability | σ^2^accession | σ^2^block | σ^2^residual |
| --- | --- | --- | --- | --- |
| RWC - WW | 0.0 | 0.03376 | 0.00152 | 0.05500 |
| RWC - WD | 0.2282 | 0.02240 | 0.00780 | 0.12100 |
| FW/DW - WW | 0.0933 | 0.80270 | 0.00775 | 0.02950 |
| FW/DW - WD | 0.8416 | 0.47500 | 0.00176 | 0.08570 |
| A - WW | 0.1198 | 0.10150 | 0.22010 | 0.17100 |
| A - WD | 0.0 | 0.01074 | 0.13320 | 0.01840 |
| E - WW | 0.2432 | 0.09763 | 0.03824 | 0.06970 |
| E - WD | 0.0 | 0.00909 | 0.02325 | 0.54400 |
| gs - WW | 0.2027 | 0.09917 | 0.00275 | 0.00815 |
| gs - WD | 0.0 | 0.01663 | 0.00140 | 0.00407 |
| C*a* - WW | 0.2655 | 0.00000 | 0.00960 | 0.12400 |
| C*a* - WD | 0.0 | 0.12270 | 0.01560 | 0.12700 |
| C*b* - WW | 0.1011 | 0.17180 | 0.00409 | 0.03890 |
| C*b* - WD | 0.0 | 0.13630 | 0.00835 | 0.05000 |
| C*cx* - WW | 0.1388 | 0.00000 | 0.00079 | 0.02910 |
| C*cx* - WD | 0.0 | 0.13160 | 0.00216 | 0.02710 |
| C*i* - WW | 0.0 | 0.00000 | 1.65000 | 1.47100 |
| C*i* - WD | 0.0 | 0.00000 | 1.85400 | 1.42400 |
| A/E - WW | 0.2009 | 0.00616 | 0.06731 | 0.05330 |
| A/E - WD | 0.6447 | 0.00415 | 0.29200 | 0.52100 |
| A/gs - WW | 0.2539 | 0.03374 | 0.76100 | 2.87000 |
| A/gs - WD | 0.0 | 0.00481 | 4.97100 | 9.76000 |
| C*a*/C*b* - WW | 0.2940 | 0.00000 | 0.00169 | 0.00956 |
| C*a*/C*b* - WD | 0.3980 | 0.00000 | 0.00147 | 0.00790 |
| C*a*+C*b* - WW | 0.1821 | 0.00000 | 0.01320 | 0.16400 |
| C*a*+C*b* - WD | 0.0 | 0.12620 | 0.02340 | 0.17200 |
| (C*a*+C*b*)/C*cx* | 0.6201 | 0.02794 | 0.00575 | 0.05340 |
| (C*a*+C*b*)/C*cx* - WD | 0.0 | 0.00000 | 0.00600 | 0.15700 |

Phenotypic traits: A – net CO_2_ assimilation rate, E – transpiration rate, gs – stomatal conductance of CO_2_, C*i* – sub-stomatal CO_2_ concentration, C*a* – chlorophyll *a* concentration, C*b* - chlorophyll *b* concentration, C*cx* – carotenes and xanthophylls concentration, RWC – leaf relative water content, FW/DW – leaf fresh: dry weight ratio, SLA – specific leaf area, LT – leaf thickness, A/E – instantaneous water use efficiency, A/gs – intrinsic water use efficiency.
