# Supplementary material for: Common bean SNP alleles and candidate genes affecting photosynthesis under contrasting water regimes
Source: Hortic Res. 2021 Jan 1;8:4. doi: 10.1038/s41438-020-00434-6 (PMC7775448; doi:10.1038/s41438-020-00434-6)
Supplement: Supplementary file 11 — Supplementary Table S7 [file 41438_2020_434_MOESM11_ESM.docx]

**Table S7** - Percentage of associated SNP variants with an effect of decrease and increase of the traits value, and maximum and minimum phenotypic variance explained by the SNPs associated with 6 photosynthesis-related traits under well-watered and water deficit conditions.

WD – water deficit conditions; WW-well-watered conditions; %SNPs_DECREASE_ – Percentage of associated SNPs for which the effect of the rare allele resulted in a decrease of the trait value when compared to the most frequent allele; %SNPs_INCREASE_ - Percentage of associated SNPs for which the effect of the rare allele results in an increase of the trait value when compared to the most frequent allele; Var_MIN_ – smallest proportion of variance explained by a significant SNP; Var_MAX_ – largest proportion of variance explained by a significant SNP; Larger SNP_EFFECT -_ Effect of variant allele explaining the largest proportion of the trait variance.

| Trait | %SNPs_DECREASE_ | %SNPs_INCREASE_ | Var_MIN_  (SNP ID; location) | Var_MAX_  (SNP ID; location) | Larger SNP_EFFECT_ |
| --- | --- | --- | --- | --- | --- |
| Net CO_2_ assimilation rate (sqrt_A)  WD, in µmol CO_2_/m^2^s | 0 | 100 | 0.0575  (DART09237; chr10: 976,191 bp) | 0.1198  (DART09339; chr10: 3,864,037bp) | (+)0.2399 |
| Net CO_2_ assimilation rate (sqrt_A)  WW, in µmol CO_2_/m^2^s | 0 | 100 | 0.0756  (SNP04396; ch10: 8,374,526 bp) | 0.1039  (SNP04526; chr10: 23,801,180 bp) | (+)0.3450 |
| Transpiration rate (sqrt_E)  WD, in mmol H_2_O/m^2^s | 0 | 100 | 0.0622  (DART09907; chr10: 39,515,883 bp) | 0.0842  (DART09339; chr10: 3,864,037bp) | (+)0.1175 |
| Transpiration rate (sqrt_E)  WW, in mmol H_2_O/m^2^s | 11.1 | 88.9 | 0.0641  (DART08825; chr09: 21,542,045 bp) | 0.1160  (SNP01992; chr05: 344,200 bp) | (+)0.1851 |
| Stomatal conductance (sqrt_gs)  WD, in mol CO_2_/m^2^s | 7.7 | 92.3 | 0.0503  (SNP04351; chr10: 3,302,998 bp) | 0.0795  (SNP04694; chr10: 39,515,883 bp) | (+)0.0366 |
| Stomatal conductance (sqrt_gs)  WW, in mol CO_2_/m^2^s | 23.8 | 76.2 | 0.0505  (DART01548; chr02: 25,698,956 bp;  DART01554; chr02: 25,812,191 bp) | 0.1200  (SNP04526; chr10: 23,801,180 bp) | (+)0.0753 |
| Chlorophyll *a* concentration (sqrt_C*a*) WD, in mg/g dry weight | 25 | 75 | 0.0577  (SNP02054; chr05: 4,808,704 bp;  SNP02055; chr05: 4,884,964 bp) | 0.1244  (SNP04635; ch10: 36,497,500 bp) | (+)0.2830 |
| Chlorophyll *a* concentration (sqrt_C*a*) WW, in mg/g dry weight | 100 | 0 | 0.0392  (SNP00315; chr01: 38,347,892 bp) | 0.0515  (SNP01123; chr03: 5,147,546 bp) | (-)0.1180 |
| Chlorophyll *b* concentration (sqrt_C*b*) WD, in mg/g dry weight | 33.3 | 66.7 | 0.0772  (SNP00315; chr01: 38,347,892 bp) | 0.1421  (SNP00345; chr01: 43,512,983 bp) | (+)0.1517 |
| Chlorophyll *b* concentration (sqrt_C*b*) WW, in mg/g dry weight | 100 | 0 | 0.0724  (SNP00315; chr01: 38,347,892 bp) | 0.0999  (SNP01123; chr03: 5,147,546 bp) | (-)0.0970 |
| Carotenoids and xantophylls concentration (sqrt_C*cx*)  WD, in mg/g dry weight | 0 | 100 | 0.0525  (SNP02054; chr05: 4,808,704 bp;  SNP02055; chr05: 4,884,964 bp) | 0.0990  (SNP04635; chr10: 36,497,500 bp) | (+)0.1189 |
| Carotenoids and xantophylls concentration (sqrt_C*cx*)  WW, in mg/g dry weight | 75 | 25 | 0.0500  (SNP01123; chr03: 5,147,546 bp) | 0.0746  (SNP01983: chr05: 150,178 bp) | (+)0.0772 |
