# Supplementary material for: Common bean SNP alleles and candidate genes affecting photosynthesis under contrasting water regimes
Source: Hortic Res. 2021 Jan 1;8:4. doi: 10.1038/s41438-020-00434-6 (PMC7775448; doi:10.1038/s41438-020-00434-6)
Supplement: Supplementary file 12 — Supplementary Table S8 [file 41438_2020_434_MOESM12_ESM.docx]

**Table S8:** MapMan functional categories (Bin names and codes) and annotation of the 95 candidate genes identified for the traits A_n_, E, gs, C*a*, C*b* and C*cx*, under well-watered (WW) and water-deficit (WD) conditions. The candidates genes located in regions where the strongest associations were detected, located in regions associated with multiple traits, and the ones we considered to have a more relevant biological function are highlighted in yellow.

| Gene ID | Bin name (Bin code)^1^ | Gene annotation^2^ | Trait |
| --- | --- | --- | --- |
| Phvul.002G128700 | not assigned (35.2) | not available | gs-WW |
| Phvul.005G002100 | not assigned.annotated (35.1) | translocase inner membrane subunit 44-2 | Ccx-WW |
| Phvul.005G004500 | RNA processing (16.11.1.1.1) | nuclear matrix protein-related | E-WW, gs-WW |
| Phvul.006G151900 | not assigned (35.2) | not available | gs-WW |
| Phvul.006G172601 | not assigned (35.2) | not available | gs-WW |
| Phvul.008G017800 | Oxidoreductase (50.1.1) | NAD(P)-linked oxidoreductase superfamily protein | gs-WW |
| Phvul.008G017900 | not assigned.annotated (35.1) | Exostosin family protein | gs-WW |
| Phvul.008G291300 | not assigned.annotated (35.1) | O-fucosyltransferase family protein | gs-WW |
| Phvul.009G046100 | not assigned.annotated (35.1) | CTC-interacting domain 11 | gs-WW |
| Phvul.009G147200 | Solute transport (24.2.1.5) | nodulin MtN21 /EamA-like transporter family protein | gs-WW |
| Phvul.010G026100 | External stimuli response (26.8.2.1) | Disease resistance protein (TIR-NBS-LRR class) family | E-WW |
| Phvul.010G049900 | not assigned.annotated (35.1) | GRAM domain family protein | gs-WW |
| Phvul.010G053300 | not assigned.annotated (35.1) | Phosphoglycerate mutase family protein | An-WW, E-WW |
| Phvul.011G216200 | Protein kinase (SCY1) (18.4.17) | Protein kinase family protein with ARM repeat domain | gs-WW |
| Phvul.001G259400 | Lipid metabolism (5.1.4.4) | NAD(P)-binding Rossmann-fold superfamily protein | E-WD, gs-WD |
| Phvul.002G008700 | Amino acid metabolism (4.1.2.2.8.6) | branched-chain amino acid aminotransferase 5 / branched-chain amino acid transaminase 5 (BCAT5) | gs-WD |
| Phvul.003G267600 | Solute transport (24.4.3) | not available | Ca-WD |
| Phvul.005G045500 | Protein homeostasis (19.4.3.1) | Eukaryotic aspartyl protease family protein | Ca-WD, Ccx-WD |
| Phvul.005G045600 | not assigned.annotated (35.1) | evolutionarily conserved C-terminal region 8 | Ca-WD |
| Phvul.005G045700 | Protein translocation, nucleoporin of nuclear pore complex (23.5.1.1.5.2) | nuclear pore anchor | Ca-WD, Ccx-WD |
| Phvul.005G045800 | not assigned.annotated (35.1) | Eukaryotic aspartyl protease family protein | Ca-WD |
| Phvul.005G045900 | not assigned.annotated (35.1) | DNA glycosylase superfamily protein | Ca-WD |
| Phvul.010G000300 | Oxidoreductase (50.1.14) | 2-oxoglutarate (2OG) and Fe(II)-dependent oxygenase superfamily protein | An-WD |
| Phvul.010G000500 | RNA biosynthesis (15.5.8) | not available | An-WD, E-WD |
| Phvul.010G001200 | not assigned.annotated (35.1) | LMBR1-like membrane protein | An-WD, E-WD |
| Phvul.010G004650 | not assigned.annotated (35.1) | glycerol-3-phosphate acyltransferase 6 | An-WD |
| Phvul.010G005000 | not assigned.annotated (35.1) | josephin protein-related | An-WD |
| Phvul.010G005100 | Lipid metabolism | sulfoquinovosyldiacylglycerol 2 | An-WD |
| Phvul.010G005200 | not assigned.annotated (35.1) | HXXXD-type acyl-transferase family protein | An-WD |
| Phvul.010G005300 | not assigned.annotated (35.1) | RING/FYVE/PHD zinc finger superfamily protein | An-WD |
| Phvul.010G005400 | not assigned.annotated (35.1) | RHO guanyl-nucleotide exchange factor 7 | An-WD |
| Phvul.010G005500 | not assigned.annotated (35.1) | Bifunctional inhibitor/lipid-transfer protein/seed storage 2S albumin superfamily protein | An-WD |
| Phvul.010G005600 | not assigned (35.2) | not available | An-WD |
| Phvul.010G005700 | not assigned (35.2) | Protein of unknown function (DUF3527) | An-WD |
| Phvul.010G022500 | not assigned (35.2) | not available | An-WD |
| Phvul.010G023500 | not assigned.annotated (35.1) | Disease resistance protein (TIR-NBS-LRR class) family | An-WD, E-WD, gs-WD |
| Phvul.010G023600 | Protein kinase (RCK) (18.4.3.7) | Protein kinase superfamily protein | An-WD |
| Phvul.010G023700 | not assigned (35.2) | Protein of unknown function (DUF760) | An-WD |
| Phvul.010G023800 | Hydrolase (50.3.2) | O-Glycosyl hydrolases family 17 protein | An-WD |
| Phvul.010G023900 | Solute transport (24.1.2.1.1) | heavy metal atpase 5 | An-WD |
| Phvul.010G024000 | not assigned.annotated (35.1) | Disease resistance protein (TIR-NBS-LRR class) family | An-WD |
| Phvul.010G024100 | External stimuli response (26.8.2.1) | Disease resistance protein (TIR-NBS-LRR class) family | An-WD |
| Phvul.010G024200 | External stimuli response (26.8.2.1) | Disease resistance protein (TIR-NBS-LRR class) family | An-WD |
| Phvul.010G024250 | External stimuli response (26.8.2.1) | Disease resistance protein (TIR-NBS-LRR class) family | An-WD |
| Phvul.010G024800 | Transcriptional regulator (26.1.2.1.2) | basic helix-loop-helix (bHLH) DNA-binding superfamily protein | An-WD |
| Phvul.010G025100 | not assigned.annotated (35.1) | NB-ARC domain-containing disease resistance protein | An-WD, E-WD |
| Phvul.010G025800 | not assigned.annotated (35.1) | Protein of unknown function (DUF506) | An-WD |
| Phvul.010G025900 | RNA biosynthesis | Mitochondrial transcription termination factor family protein | An-WD |
| Phvul.010G026100 | External stimuli response (26.8.2.1) | Disease resistance protein (TIR-NBS-LRR class) family | An-WD, E-WD |
| Phvul.010G028000 | External stimuli response (26.8.2.1) | Disease resistance protein (TIR-NBS-LRR class) family | An-WD, E-WD |
| Phvul.010G028101 | External stimuli response (26.8.2.1) | Disease resistance protein (TIR-NBS-LRR class) family | An-WD, E-WD |
| Phvul.010G028400 | External stimuli response (26.8.2.1) | Disease resistance protein (TIR-NBS-LRR class) family | An-WD |
| Phvul.010G028500 | External stimuli response (26.8.2.1) | Disease resistance protein (TIR-NBS-LRR class) family | An-WD |
| Phvul.010G028600 | not assigned.annotated (35.1) | Disease resistance protein (TIR-NBS-LRR class) family | An-WD |
| Phvul.010G028700 | External stimuli response (26.8.2.1) | Disease resistance protein (TIR-NBS-LRR class) family | An-WD |
| Phvul.010G028800 | External stimuli response (26.8.2.1) | Disease resistance protein (TIR-NBS-LRR class) family | An-WD |
| Phvul.010G028900 | External stimuli response (26.8.2.1) | Disease resistance protein (TIR-NBS-LRR class) family | An-WD |
| Phvul.010G029000 | External stimuli response (26.8.2.1) | Disease resistance protein (TIR-NBS-LRR class) family | An-WD |
| Phvul.010G029100 | External stimuli response (26.8.2.1) | Disease resistance protein (TIR-NBS-LRR class) family | An-WD |
| Phvul.010G029201 | External stimuli response (26.8.2.1) | Disease resistance protein (TIR-NBS-LRR class) family | An-WD |
| Phvul.010G029400 | not assigned.annotated (35.1) | Disease resistance protein (TIR-NBS-LRR class) family | An-WD |
| Phvul.010G029500 | not assigned.annotated (35.1) | not available | An-WD |
| Phvul.010G029600 | External stimuli response (26.8.2.1) | Disease resistance protein (TIR-NBS-LRR class) family | An-WD |
| Phvul.010G029700 | External stimuli response (26.8.2.1) | Disease resistance protein (TIR-NBS-LRR class) family | An-WD |
| Phvul.010G029800 | External stimuli response (26.8.2.1) | not available | An-WD, E-WD |
| Phvul.010G029900 | not assigned.annotated (35.1) | carboxyl terminus of HSC70-interacting protein | An-WD |
| Phvul.010G030000 | Solute transport (24.2.2.1.6) | sugar transporter 14 | An-WD |
| Phvul.010G030100 | Transferase (50.2.7) | AGC (cAMP-dependent, cGMP-dependent and protein kinase C) kinase family protein | An-WD |
| Phvul.010G030200 | RNA biosynthesis (15.1.6.2) | RNA polymerase II, Rpb4, core protein | An-WD |
| Phvul.010G030300 | Solute transport | sugar transporter 1 | An-WD |
| Phvul.010G030500 | not assigned.annotated (35.1) | auxin-responsive family protein | An-WD |
| Phvul.010G030600 | not assigned.annotated (35.1) | lipase class 3 family protein | An-WD |
| Phvul.010G030700 | Vesicle trafficking (22.6.3.7) | exocyst complex component sec15A | An-WD |
| Phvul.010G030800 | Protein biosynthesis (17.2.3) | asparaginyl-tRNA synthetase 2 | An-WD |
| Phvul.010G030900 | not assigned.annotated (35.1) | Homeodomain-like superfamily protein | An-WD |
| Phvul.010G031000 | Phytohormone action (11.10.2.3.1) | stomagen | An-WD |
| Phvul.010G031100 | Hydrolase (50.3.6) | not available | An-WD |
| Phvul.010G031200 | not assigned (35.2) | not available | An-WD |
| Phvul.010G031300 | not assigned (35.2) | not available | An-WD |
| Phvul.010G031500 | Cytoskeleton organisation (20.2.2.4) | Actin binding Calponin homology (CH) domain-containing protein | An-WD |
| Phvul.010G031600 | not assigned.annotated (35.1) | DNA binding;ATP binding | An-WD |
| Phvul.010G031700 | Cytoskeleton organisation (20.3) | Spc97 / Spc98 family of spindle pole body (SBP) component | An-WD, E-WD |
| Phvul.010G031800 | not assigned.annotated (35.1) | DNA binding;ATP binding | An-WD |
| Phvul.010G031900 | Transferase (50.2.7) | receptor-like protein kinase 1 | An-WD |
| Phvul.010G032000 | Transferase (50.2.7) | receptor-like protein kinase 1 | An-WD |
| Phvul.010G032100 | Protein biosynthesis (17.1) | Ribosomal protein S6e | An-WD, E-WD |
| Phvul.010G032700 | Vesicle trafficking (22.6.3) | Sec23/Sec24 protein transport family protein | An-WD, E-WD |
| Phvul.010G040500 | Cell wall organisation (21.9.1) | Fatty acid hydroxylase superfamily | An-WD, E-WD |
| Phvul.010G040600 | Cell wall organisation (21.9.1) | Fatty acid hydroxylase superfamily | An-WD, E-WD |
| Phvul.010G040700 | Vesicle trafficking (22) | not available | An-WD, E-WD |
| Phvul.010G105900 | not assigned.annotated (35.1) | glycosyl transferase family 1 protein | Ca-WD, Ccx-WD |
| Phvul.010G118300 | Transferase/SNF1-related protein kinase (SnRK3) (50.2.7) | CBL-interacting protein kinase 9 | gs-WD |
| Phvul.010G124400 | Protein homeostasis (19) | Subtilase family protein | An-WD, E-WD, gs-WD |
| Phvul.010G125000 | Multi-process regulation (27.5.3) | Phosphoinositide phosphatase family protein | An-WD, E-WD, gs-WD |
| Phvul.010G125200 | Protein biosynthesis (17) | CCAAT-binding factor | E-WD, gs-WD |

^1^Bin names and codes retrieved from MapMan v4.2.0

^2^Information available in the file “Pvulgaris_442_v2.1.annotation_info.txt” (*Phaseolus vulgaris* v*2*.1, U.S.A. Department of Energy Joint Genome Institute, Phytozome v12.0: <http://phytozome.jgi.doe.gov/>)
